# Chemical Recycling of Flexible Polyurethane Foams by Aminolysis to Recover High-Quality Polyols

**DOI:** 10.1021/acssuschemeng.3c02311

**Published:** 2023-07-10

**Authors:** Maja Grdadolnik, Blaž Zdovc, Ana Drinčić, Ozgun Can Onder, Petra Utroša, Susana Garcia Ramos, Enrique Dominguez Ramos, David Pahovnik, Ema Žagar

**Affiliations:** † Department of Polymer Chemistry and Technology, National Institute of Chemistry, Hajdrihova 19, Ljubljana SI-1000, Slovenia; ‡ Intermediates Technical Service & Development department, Repsol Quimica S.A., Mendez Álvaro 44, CP28045 Madrid, Spain

**Keywords:** sustainable chemistry, waste prevention, chemical recycling, aminolysis, flexible polyurethane foam, microwave chemistry

## Abstract

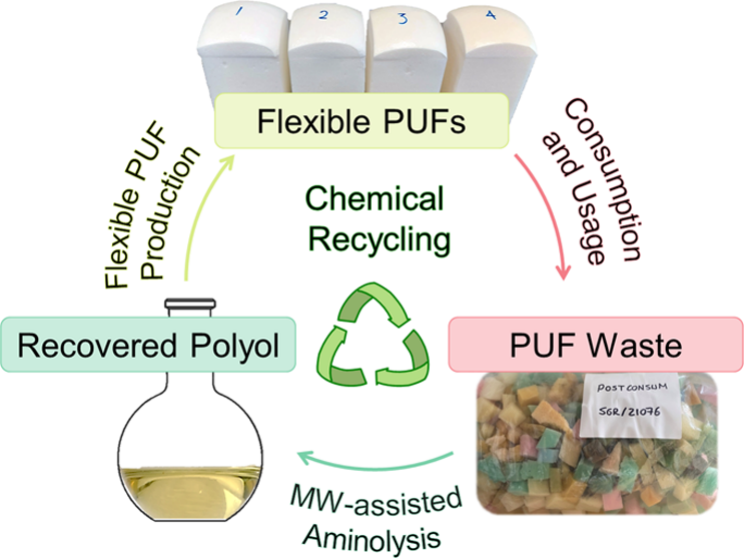

Polyurethane foams (PUFs) are widely used commodity materials, but most of them end up in landfills at the end of their life, which is not in line with the circular economy approach. Here, we introduce microwave-assisted aminolysis with amine reagents that contain primary and tertiary amino groups in the structure. These reagents enable complete degradation of the urethane groups in the structure of the flexible PUFs with a much lower amount of degradation reagent than is typically required for solvolysis reactions. The purified, recovered polyols are close equivalents to the corresponding virgin polyols in terms of their structural and molar mass characteristics. Therefore, they can be used for the production of high-quality PUFs without having to adapt the synthesis process. The flexible PUFs made from recovered polyols have comparable mechanical properties to those made from virgin polyols.

## Abstract 2

Close analogues of virgin polyether polyols were recovered from polyurethane foams with reagents containing primary and tertiary amino groups in the structure.

## Introduction

Plastic materials are key components of almost every technology today. The production of plastics consumes substantial feedstock resources, and after their service life, they represent a waste that has become a growing environmental problem in terms of pollution and related climate change due to inadequate circular economy.^1^ Chemical recycling of polymers is an alternative to traditional methods of treating plastic waste (landfill, incineration, mechanical recycling) by converting them into feedstock suitable for re-polymerization into the same or new materials.^2−4^ Chemical recycling is particularly suitable for polymers with chemically labile groups in the backbone and is usually performed by solvolysis using nucleophilic solvents in the presence of a catalyst.^5−8^

Polyurethanes are the sixth most produced polymer, with polyurethane foams (PUFs) accounting for about 67% of global polyurethane consumption.^9,10^ The PUF market is forecast to increase from 15 million tons in 2020 to 20 million tons in 2025 as mattress, furniture, electronics, automotive, and construction industries grow.^11^ Consequently, both the consumption of fossil resources for the production of polyols and diisocyanates, and the amount of PUF waste are expected to increase. Therefore, there is a clear need for alternative solutions to sustain growth projections for PUF production and prevent waste accumulation.

Since pyrolysis and mechanical recycling are not the most suitable methods for treating PUF waste due to environmental issues and the cross-linked structure, respectively, chemical recycling has recently become a very interesting research topic.^9,12−14^ Chemical recycling of PUFs made from polyether polyols is based on the cleavage of the urethane bonds, leaving the ether groups in the polyether polyol intact. Depending on the mechanism of urethane bond degradation, recycling technologies are mainly classified into hydrolysis,^15,16^ glycolysis,^17−34^ alcoholysis,^35^ acidolysis,^7,36−40^ aminolysis,^41−48^ and hydrogenation.^49,50^ To achieve efficient degradation of urethane groups, the process of PUF recycling must be carried out at high temperatures (200 °C and above) by heating the reaction mixtures by conventional heating or, less commonly, by microwaves (MWs), which allows an equally effective degradation process in a much shorter time.^7,20,26,40,42^

PUFs can be efficiently degraded by hydrolysis,^15,16^ alcoholysis with *tert*-amyl alcohol,^35^ or catalytic hydrogenation,^49,50^ but these reactions are not selective for the degradation of only the urethane groups, as the urea groups of the hard segments in the PUF structure also completely degrade, resulting in a mixture of recycled polyols and undesired aromatic amines (e.g., toluene diamine, TDA, or methylene diphenyl diamine, MDA). Glycolysis is the most studied chemical recycling method for polyurethane (PU) waste and involves transcarbamoylation of the urethane bonds. However, thermal degradation of PU is not excluded at high reaction temperatures.^23,24,28,31,32^ Glycolysis is preferred over hydrolysis because the reaction conditions are milder and fewer toxic aromatic amine side products are formed. Glycolysis can be performed as a single-phase^17−24^ or split-phase process.^25−34^ It has been shown that the quality of the reaction product depends on the reaction temperature and time, the type of catalyst and glycol used, the weight ratio of PUF to glycol, and the size of the PUF particles introduced into the reactor. Single-phase glycolysis leads to complex mixtures that have been used for the synthesis of PU elastomers,^21−23^ coatings and adhesives,^18^ reaction injection molding PU materials,^17,18^ and rigid PUFs.^19,20^ Split-phase glycolysis enables the recovery of high-quality polyols, but despite their purification, high-performance flexible PUFs cannot be synthesized exclusively from glycolysis-derived recycled polyols (RPs),^29,30,32,33^ which is attributed to the contamination of RPs with glycol medium, glycolysis catalyst, and/or other side products. Acidolysis of PUFs with dicarboxylic acids to produce RPs can be performed with small amounts of the degradation reagent since the reaction is irreversible and leads to the formation of a more thermally stable amide bond after decarboxylation of the formed carbamic acid. Moreover, the carboxyl groups of the acid reagent may react with aromatic amines, acting as an amine scavenger. Nevertheless, acidolysis cannot produce RPs that would be a close equivalent of virgin polyols (VPs) in terms of functionality^7^ since at low amounts of the acid reagent used, the degradation of the urethane bonds is not complete, leading to the aromatic amino-functionalized RPs, whereas at higher amounts of the acid reagent used, the extent of esterification between the hydroxyl groups of the polyol and the carboxyl groups of the reagent is more pronounced, leading to the polyol chains terminated with carboxyl groups. Both the amino and especially the carboxyl end groups of the polyol significantly influence the quality and performance of the flexible PUFs synthesized from acidolysis-derived RPs.^7^ A less studied PUF degradation process is aminolysis, which can be carried out in the presence (triazabicyclodecene/methanesulfonic acid mixture or alkali hydroxides)^41−45^ or absence of a catalyst.^45−48^ In aminolysis, the polyol in the urethane group is exchanged with the amine, which can be aliphatic diamines or polyamines with primary and, in some cases, also secondary amino groups in the structure,^41,42,44−47^ or alkanolamines.^41,43,45,48^ Depending on the reaction conditions, the result of PUF aminolysis is either a complex mixture produced in a single-phase process that is suitable for less demanding applications only (e.g., curing agents for epoxy resins,^47^ polyurethane-urea coatings),^42^ or a polyether polyol, which is produced in a split-phase process^43^ and is of poor quality and only suitable for the synthesis of rigid PUFs.^12^ The exact functionality, molar mass characteristics, and detailed characterization of aminolysis-derived RPs are not reported.^43,44,48^

Existing methods of chemical recycling of PUF waste suffer from incomplete degradation of urethane groups and lack of selectivity for degradation of urethane bonds alone, resulting in differently functionalized polyols containing various side products.^33^ For this reason, RPs are used only as partial replacements of VPs in formulations for the production of new flexible PUFs.^7,11,29,30,32,33^ The aim of our work was to develop an efficient recycling process for flexible PUFs in order to produce RPs that are a close equivalent of VPs in terms of functionality, molar mass, and purity to enable their re-polymerization into new flexible PUFs of the same quality and performance as those produced from VPs.

## Experimental Section

### PUF Degradation

PUFs were cryogenically ground into powder using a vibratory ball mill (Tehtnica Millmix 20 Domel, Slovenia) to produce PUF particles with sizes in the range of several millimeters. The reaction mixture was prepared by homogeneously mixing the amine degradation reagent; i.e., tris(2-aminoethyl)amine (TREN), hyperbranched poly(ethylenimines) with number-average molar masses of 600 and 1800 g mol^–1^ (PEI-600 and PEI-1800), or hexamethylenediamine (HMDA) with or without triethylenediamine (DABCO) catalyst, and the polyol medium (VP or RP), followed by addition of ground PUF to this mixture. The weight ratio of PUF to polyol medium was 2:1 (6/3 g) and was kept constant. The presence of medium prevents foam caking and ensures efficient stirring of the reaction mixtures by a magnetic stirrer. The reaction mixture was transferred to a 30 mL glass reactor vessel with a magnetic stirrer and then sealed tightly with PTFE-coated silicone septum. Prior to degradation, the reaction mixtures were purged with nitrogen to avoid darkening of the degradation products due to oxidation processes.^7,51^ The reaction mixtures were heated with MWs in a laboratory microwave reactor Monowave 400 (Anton Paar GmbH, Austria). The initial mixture was a thick paste requiring a preheating step (heating to 175 °C in a period of 3 min) to partially degrade the PUF and allow efficient stirring in the main reaction step. Prior to the main heating step, the reaction mixture was homogenized manually. The main heating cycle consisted of heating the reaction mixture to a predetermined temperature (210, 220, or 230 °C) in a span of 5 min and maintaining this temperature for a specified time (15, 30, or 40 min). After completion of the reaction, the vials were cooled to 70 °C by a stream of compressed air. Aminolysis of PUF5611 was also carried out in bulk without the use of a medium; however, in this particular case, two preheating cycles (heating to 175 °C in 3 min) were necessary to partially degrade the foam and allow sufficient stirring in the main heating cycle. The resulting reaction mixtures were centrifuged at 9000 rpm for 10 min to separate the upper polyol phase (the so-called crude polyol) from the lower solid phase (residues of the PUF hard segments). In the case of two-step aminolysis, the first step was carried out with TREN or HMDA at 220 °C for 30 min as described above, while in the second step, TREN was added to a crude polyol in excess with respect to the content of residual urethane groups in the RP as determined by ^1^H NMR. The second step of aminolysis was carried out at 220 °C for 20 min.

### Purification of RPs

Crude polyol dissolved in ethyl acetate (EtOAc; *c* = 1 g mL^–1^) was purified by liquid–liquid extraction with acidic water (0.1 M HCl) followed by water to remove amino-functionalized low-molar-mass side products (mainly aromatic diamines). EtOAc and water were removed from the purified RP by rotary evaporation at 60 °C. The lower solid phase obtained after centrifugation of the reaction mixture and decantation of crude RP consists of residues of PUF hard segments, functionalized with amino groups or the amino reagent used, to which some remaining RP and TDA are adsorbed. RP was isolated from the hard segments by dissolving it in EtOAc, and afterward it was purified in a similar manner as described above.

## Results and Discussion

**Scheme 1:**
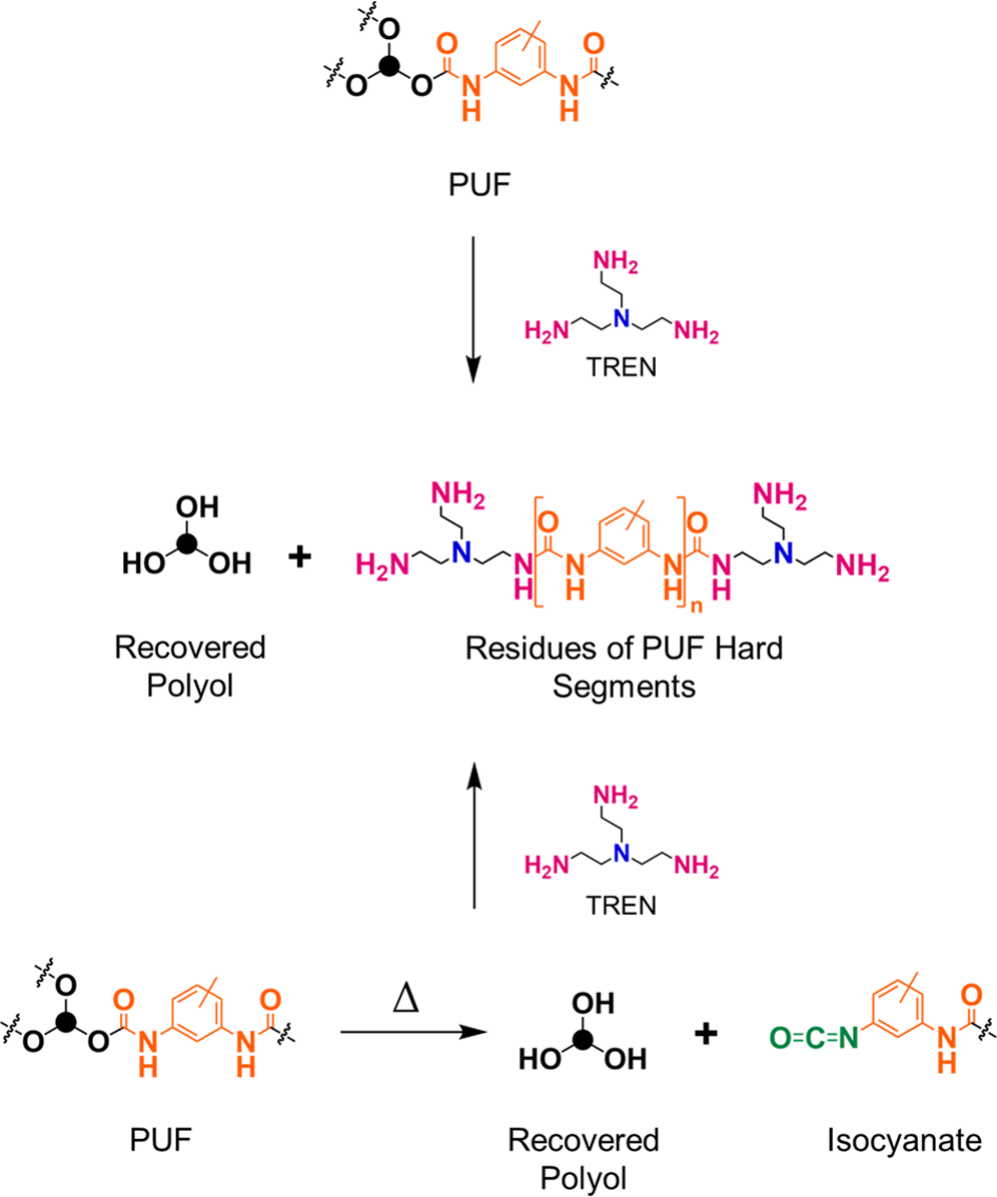
Schematic Representation of the Aminolysis of PUF with TREN and the Reaction of TREN with the Thermally Released Isocyanate Group Both reactions lead to the same product.

The degradation of PUFs with nucleophilic reagents that exchange the polyol in the urethane group usually occurs at high temperatures (up to 230 °C), where some role of thermal degradation of the urethane groups is also expected^23,24,28,31,32,36−38,42^ since they are thermally degraded in the same temperature range.^52,53^ The thermal degradation of the urethane group leads to the release of the hydroxyl and isocyanate precursors (Scheme 1). Due to the high reactivity of the isocyanate groups, they react further with the nucleophiles present in the reaction system. Since the reactivity of isocyanates with amines to form urea is higher than that of isocyanates with the hydroxyl groups of the polyol back to the urethane group,^54,55^ we chose aminolysis as the method for PUF degradation. To improve the selectivity of the amine with the thermally released isocyanate group, we used amine reagents that additionally contain a tertiary amino group in the structure (TREN and PEI), which is known to activate the isocyanate group and increase the rate of nucleophilic addition of compounds with active hydrogen to the C=N bond.^54,55^

### Aminolysis of PUFs with TREN as a Function of Temperature and Time

**Figure 1:**
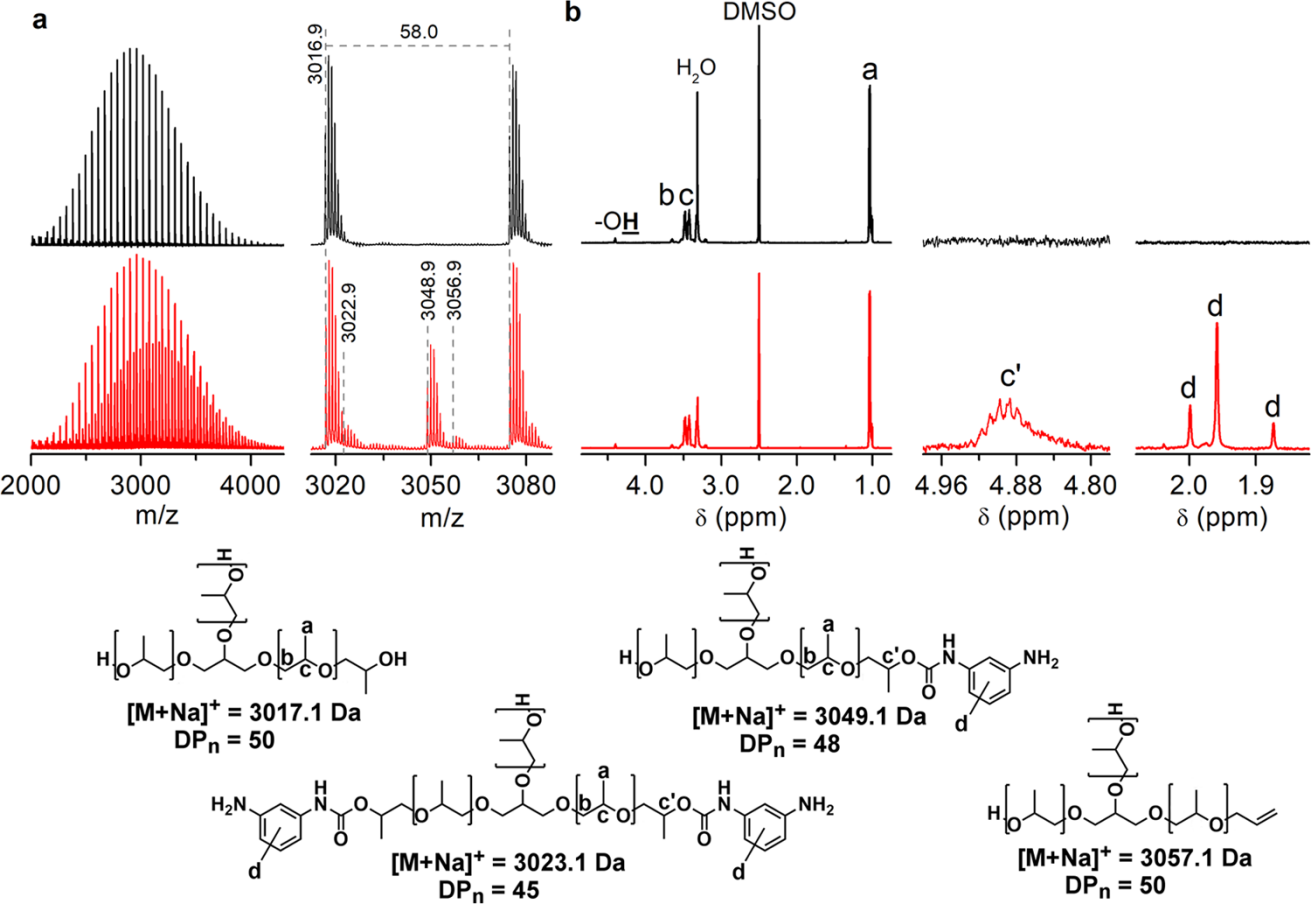
(a) MALDI-TOF mass spectra and (b) ^1^H NMR spectra with proton assignation for a typical RP (bottom) after incomplete urethane group degradation and the corresponding virgin polyol VP5611 (top). The measured monoisotopic masses of four peak populations in the MALDI-TOF mass spectrum of RP are in good agreement with the theoretical monoisotopic masses given under the proposed structures with the corresponding degrees of polymerization (DP*_n_*).

In aminolysis experiments with TREN, containing three primary amino groups and one tertiary amino group in the structure, and poly(propylene oxide) (PPO)-based PUF, a slight excess of amino per urethane group was used (1.25 equiv). Reactions were performed at different temperatures and different times. The crude RPs were isolated by pouring them from the centrifuged reaction mixtures and analyzed by matrix-assisted laser desorption/ionization time-of-flight mass spectrometry (MALDI-TOF MS), ^1^H NMR, and SEC/UV-MALS-RI. The typical MALDI-TOF mass spectrum of RP after incomplete degradation of the urethane groups in the PUF structure shows four peak populations (Figure 1a), the intensity of which depends on the degree of urethane group degradation. The main peak series corresponds to the desired hydroxyl-functionalized polyol, while the three smaller peak populations show the polyol chains terminated at one or two chain ends by the aromatic amino group due to the incomplete urethane group degradation, and by an allyl group. The undesirable allyl-terminated polyol chains, which are inactive for polymerization with diisocyanates, are formed by thermal degradation of the urethane group by decarboxylation due to an insufficient amount of added amine reagent, leading to an equilibrium between the urethane group and its isocyanate and hydroxyl precursors, which favors this slower irreversible pathway of thermal degradation of the urethane group.^42,52,53^

Typical signals of differently functionalized polyol chains are visible in the ^1^H NMR spectra of the purified polyols at 5.08 and 5.22 ppm for the allyl group, 4.40 ppm for the hydroxyl group, and at 1.87, 1.96, and 2.00 ppm for the methyl groups of three isomeric aromatic amino end groups of TDA moiety attached to the polyol via the urethane group (signals labeled (d) in Figure 1b), which are only visible when the spectra are magnified. The content of residual urethane groups in the RPs was determined from the signal intensity of the polyol methyne protons adjacent to the residual urethane groups at δ 4.88 ppm (signal indicated by (c′), Figure 1b) according to eq S1, while the TDA content in crude RPs was determined from the signal intensities of the methyl groups of the TDA isomers (δ 1.79 for 2,6-TDA and 1.88 ppm for 2,4-TDA) according to eq S2. With increasing temperature and time, the content of remaining urethane groups in the RPs decreases up to 8 mol %, with a concomitant increase in TDA content up to 5.4 wt %, but none of the reaction conditions leads to complete urethane group degradation (Table S2).

In the same order, a continuous decrease in the ratio of UV to RI signals of RPs is observed in the SEC chromatograms (Figure S1), which is due to the decreasing fraction of polyol species containing UV-active aromatic amino end groups as a result of incomplete urethane group degradation. The average molar mass of RP as determined by SEC/MALS-RI decreases with longer reaction time and increasing temperature due to the higher degree of degradation of the urethane groups (Table S2). At high degrees of degradation of urethane groups (>90%), the molar mass characteristics of RPs are comparable to those of the corresponding VP, since the contribution of a small amount of aromatic amino end groups to the total molar mass of the polyol is negligible. At larger elution volumes, SEC chromatograms of crude RPs show the presence of UV-active side products, i.e., mainly TDA at 20.9 mL and TDA derivatives, which are soluble in RPs (Figure S1). The increase in the peak area of TDA in SEC-UV chromatograms of RPs corresponds to the increasing TDA content in RPs as determined by ^1^H NMR (Table S2). The side products soluble in crude RPs were removed by purification using liquid–liquid extraction.

### Aminolysis as a Function of the Amount and Type of Amine Reagent

**Figure 2:**
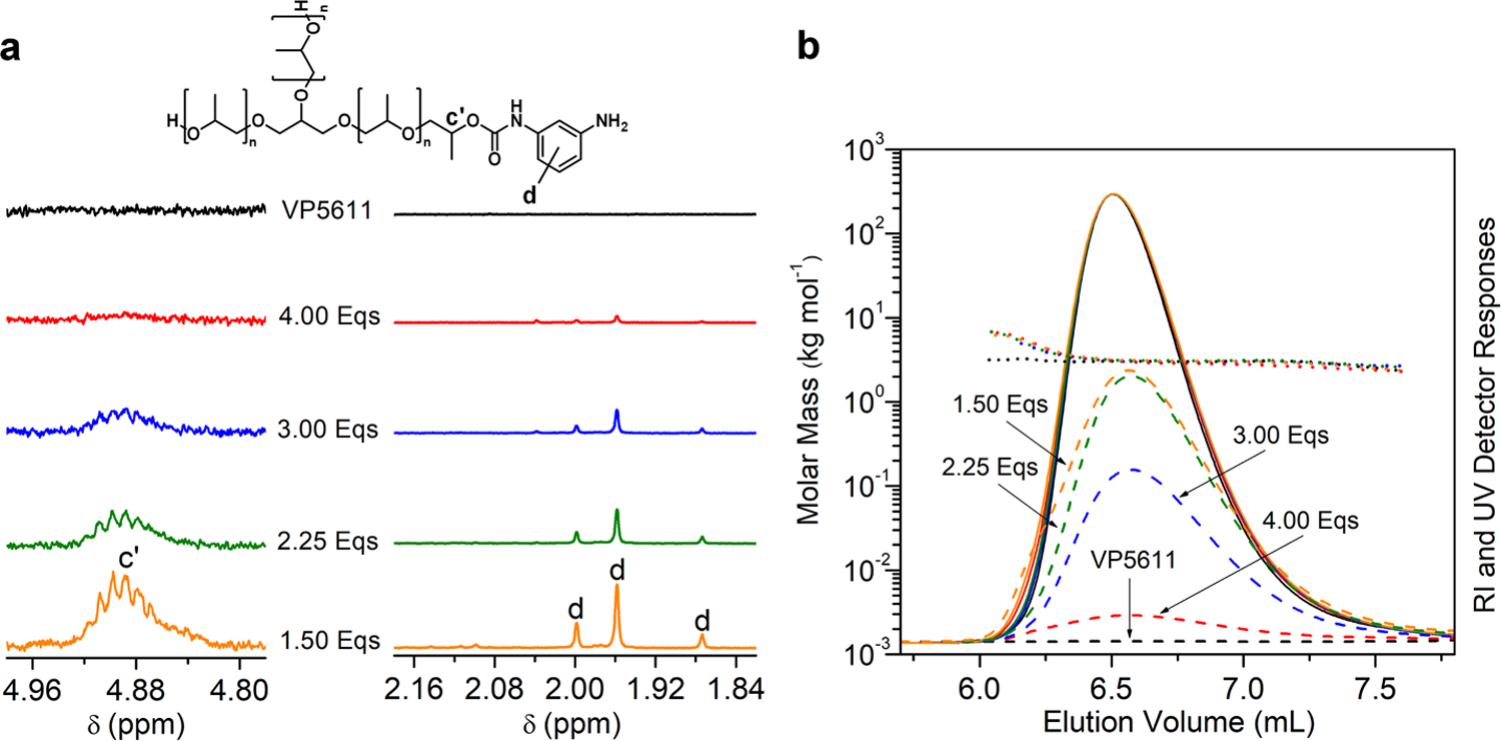
(a) Magnified ^1^H NMR spectra and (b) SEC/UV-RI chromatograms of VP5611 and RPs recovered from PPO-based PUF by aminolysis with different amounts of TREN. The solid and dashed curves in the SEC/UV-RI chromatograms represent the RI and UV detector responses, respectively, while the dotted lines show the molar mass as a function of elution volume.

To improve the degradation of urethane groups and shift the thermal equilibrium toward the desired formation of aliphatic urea, the aminolysis of PUF was performed with higher amounts of TREN (i.e., amino per urethane group from 1.50 to 4.00 equiv, corresponding to TREN per PUF from 4.8 to 13.2 wt %; Table 1; entries 1–4). The reactions were performed at 220 °C for 30 min. The undesirable allyl-functionalized polyol was not detected by ^1^H NMR or by MALDI-TOF MS at a ratio of amino to urethane groups of 2.25 and above. With increasing amounts of TREN from 1.50 to 4.00 equiv amino per urethane group, the content of remaining urethane groups in the RP decreases from 9.7 to 1.0 mol %, but with a concomitant increase in released TDA from 3.9 to 10.2 wt % (Table 1; entries 1–4). Due to the more efficient degradation of the urethane groups at higher TREN amounts, the proportion of aromatic amino end groups of the polyol decreases, which can be seen from the decreasing intensity of ^1^H NMR signal d in Figure 2a and the decreasing intensity of UV signal below the polyol peak in SEC/UV-RI chromatograms of RPs in Figure 2b.

**Table 1:** Reaction Conditions and Properties of RPs Recovered from PUFs by Aminolysis with Different Amounts of TREN and HMDA at 220 °C for 30 min^a^^,^^b^

		amino/urethane group ratio in equivalents										
entry	PUF type	TREN	HMDA	PUF/VP/reagent (g/g/g)	reagent/PUF (wt %)	urethane group content (mol %)	amino end group content (mol %)	TDA content (wt %)	allyl group	*M*_w_ (kg mol^–1^)	*Đ*	yield (%)
1	PUF5611	1.50		6/3/0.29	4.8	9.7	8.4	3.9	yes	3.2	1.03	c
2	PUF5611	2.25		6/3/0.44	7.3	5.8	5.8	6.0	no	3.1	1.02	66
3	PUF5611	3.00		6/3/0.58	9.7	3.7	3.6	7.7	no	3.1	1.02	74
4	PUF5611	4.00		6/3/0.79	13.2	1.0	0.9	10.2	no	3.0	1.02	85
5	PUF5611		4.00	6/3/0.92	15.3	7.3	6.9	7.1	no	3.1	1.02	c
6	PUF5611		4.00	6/3/0.92 + 0.30 DABCO	15.3	3.7	3.5	7.1	no	3.0	1.02	c
7^d^	PUF4811	4.00		6/3/0.68	11.3	e	0.6	9.3	no	3.5	1.02	c
8^d^	Post-consumer PUF4811	4.00		6/3/0.68	11.3	e	0.2	10.1	no	3.5	1.02	86
9^d^	PUF2832	4.00		6/3/0.56	9.3	e		13.4	no	6.0	1.08	c
10	PUF5611 from RP	4.00		6/3/0.79	13.2	e	0.7	9.5	no	3.0	1.02	83
11	PUF5611	4.00		6/3(RP)/0.79	13.2	e	0.6	10.9	no	3.0	1.01	c
12^f^	PUF5611	4.00		6/0/0.79 (bulk)	13.2	e	0.4	15.7	no	3.0	1.02	c

a Molar mass characteristics of VPs as determined by SEC/MALS-RI are *M*_w_ = 3.0 kg mol^–1^, *Đ* = 1.02 for VP5611; *M*_w_ = 3.5 kg mol^–1^, *Đ* = 1.02 for VP4811; and *M*_w_ = 6.0 kg mol^–1^, *Đ* = 1.06 for VP2832.

b The contents of urethane groups, TDA, and amino end groups in RP were calculated according to eqs S1–S3, respectively.

c Not determined.

d The molar masses for the copolymer polyols VP4811 and VP2832 and their chemical composition were considered in the calculations of the contents of urethane and amino groups, and the TDA content.

e The intensity of the polyol ^1^H NMR methyne signal near the urethane group is too low for accurate quantification.

f Aminolysis of PUF performed in bulk.

With increasing TREN amount, the isolation of RP is facilitated, since the final reaction mixtures are dispersions at low amounts of TREN used, whereas at higher TREN amounts (≥7.3 wt %), a two-phase system is obtained consisting of a liquid polyol upper phase and the residual solid hard segments (Figure S3). Reaction mixtures, especially those obtained with low TREN amounts, need to be centrifuged to separate the liquid polyol from the solid hard segments so that RP can be decanted from the reaction mixture.

To identify the importance of the presence of both primary and tertiary amino functional groups in the structure of the same reagent molecule, the degradation of PPO-based PUF with HMDA, containing only primary amino groups in the structure, was studied in the presence and absence of a tertiary amine catalyst, i.e., DABCO, commonly used in PUF synthesis (Table 1; entries 5–6). The degradation of PUF with HMDA at 4.00 equiv of amino per urethane group (15.3 wt % HMDA per PUF) results in 7.3 mol % remaining urethane groups in RP, while the value for TREN at the same ratio of primary amino per urethane group is 1.0 mol % (Figure S4). Compared to aminolysis of PUF with HMDA only, the additional use of DABCO catalyst improves the urethane group degradation, but RP still contains more residual urethane groups than RP recovered from PUF with TREN (3.7 vs 1.0 mol %), at the same ratios of primary and tertiary amino per urethane group (4.00 and 1.36 equiv, respectively). This shows the importance of the presence of primary and tertiary amino groups in the structure of the same degradation agent.

Aminolysis of PUF was also performed using hyperbranched PEIs with number-average molar masses of 600 and 1800 g mol^–1^ (Table S3). Hyperbranched PEIs contain primary and tertiary amino groups as well as secondary amino groups in the structure. After aminolysis of PUF at 220 °C for 30 min with 11.0 wt % PEI-600 per PUF (3.0 equiv of amino groups per urethane group), the degradation of urethane groups was almost complete, as only 1.1 mol % urethane groups were detected by ^1^H NMR in the RP. Efficient degradation of the urethane groups in the PUF structure is evident also from SEC/MALS-UV and FTIR results of purified RPs (Figure S5). However, the formation of allyl-functionalized polyol was completely prevented only when 14.2 wt % PEI-600 per PUF was used. The more pronounced formation of allyl-functionalized polyol during aminolysis with PEI was attributed to its higher molar mass compared to TREN and consequently to its lower mobility. This explanation was confirmed by higher-molar-mass PEI-1800, which did not completely prevent the formation of allyl-functionalized polyol even when 14.8 wt % PEI-1800 per PUF was used.

The optimal aminolysis process using TREN or PEI-600 was also applied to the recycling of post-industrial and post-consumer PUF wastes containing unknown additives such as dyes and fillers. These PUFs were prepared from a commonly used copolyether three-arm-star polyol with ethylene oxide (EO) and propylene oxide (PO) repeating units attached to a glycerol core. The results in terms of residual urethane group content and TDA content in the RPs are comparable to the values obtained for the PPO-based PUF (Table 1; entries 7–8 and Table S3; entries 4–5). Furthermore, the molar mass characteristics of the RPs are comparable to those of the corresponding VP, indicating successful urethane group degradation in the structure of the copolyether polyol-based PUFs (Figure S6). The optimal aminolysis procedure was also successful in recovering the copolyether polyol from the methylene diphenyl diisocyanate (MDI)-based PUF (Table 1; entry 9 and Figure S7).

Finally, the PUFs synthesized from 100% RP5611 were again subjected to an aminolysis process with TREN and PEI-600 under optimal experimental conditions (Table 1; entry 10, Table S3; entry 6). The structural properties and molar mass characteristics of the twice-recycled RPs are comparable to those of the corresponding VPs (Figure S8), showing that PUFs can be recycled at least twice using this process.

Typical isolation of RP from the centrifuged reaction mixture includes decanting of the RP from the reaction mixture. The RP adsorbed onto the hard segments was isolated by extraction into ethyl acetate. The thus-obtained RP yields were higher than theoretically possible (taking into account the recovered polyol from PUF and polyol medium), mainly due to the contamination of the polyol with TDA. After purification of the crude polyol dissolved in EtOAc by liquid–liquid extraction with acidified water followed by water, the yield depends on the degree of degradation of the urethane groups (Tables 1 and S3). At a degree of urethane group degradation of more than 99%, the typical yield of the recovered polyol was 83–86%, based on the theoretical yield. In contrast, at lower degrees of degradation of the urethane groups in the PUF structure, the polyol yield after purification was lower, mainly due to gel formation during liquid–liquid extraction at the water/EtOAc interface as a result of protonation of the aromatic amino end groups of the polyol, which causes poorer phase separation.

### Two-Step Aminolysis

**Figure 3:**
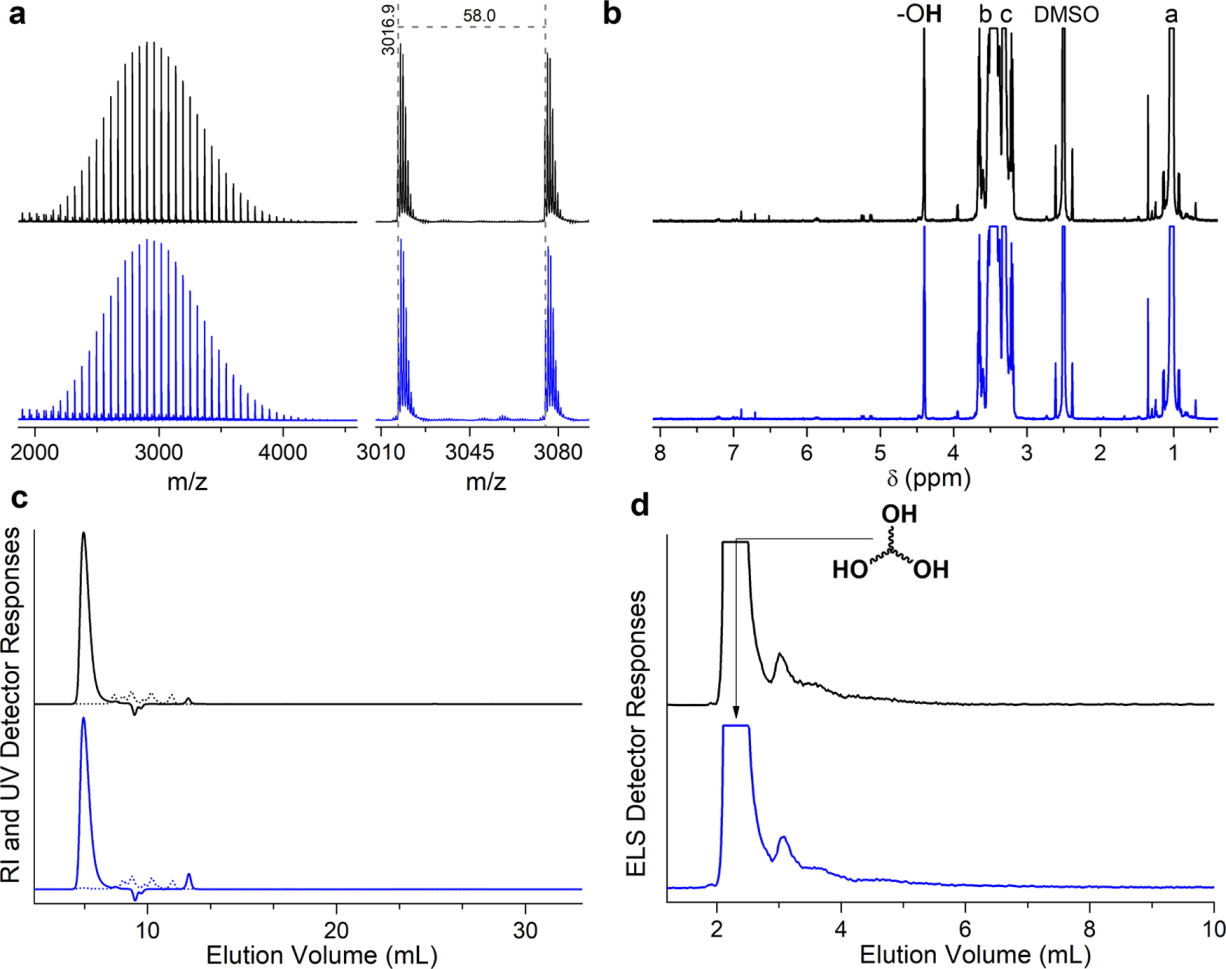
Comparison of results of (a) MALDI-TOF MS, (b) ^1^H NMR, (c) SEC/RI-UV, and (d) liquid adsorption chromatography coupled to evaporative light scattering detector of purified RP recovered from PPO-based PUF by a two-step aminolysis procedure with TREN (blue) and the corresponding VP5611 (black). The solid and dashed curves in (c) represent the RI and UV detector responses, respectively.

Previous results show that a 4-fold excess of amino per urethane group is required to achieve almost complete (≥99%) degradation of urethane groups in the structure of PUFs. To reduce the amount of reagent, aminolysis with TREN was performed in two steps. In the first step, only 7.3 wt % TREN per PUF was used (amino per urethane group of 2.25), an amount that prevented the formation of allyl-functionalized polyol and resulted in 5.8 mol % remaining urethane groups after 30 min at 220 °C (Table 2; entry 1a). Subsequently, 6 g of the crude RP thus obtained was subjected to the second aminolysis cycle, in which the 1.2 wt % of reagent was added, corresponding to a 4-fold excess of TREN-amino per remaining urethane groups of the RP (Table 2; entry 1b). In this way, complete degradation of urethane groups without formation of allyl-functionalized polyol chains was achieved with 34% less TREN compared to the one-step aminolysis procedure as shown by the structural characterization of purified RP by MALDI-TOF MS, ^1^H NMR, SEC/RI-UV, and HPLC, only the degradation time was extended by 20 min (Figure 3). In addition, two-step aminolysis results in RP containing a slightly lower amount of TDA than RP obtained by one-step aminolysis because a lower amount of reagent was used in the first step. Similar results were obtained for the two-step aminolysis of copolyether polyol-based PUF4811 (Table 2; entries 2a and 2b and Figure S13). Alternatively, TREN may be replaced in the first aminolysis step by HMDA (15.3 wt %; 4.0 amino groups per urethane group; Table 2; entry 3a). Complete degradation of the urethane groups is then achieved in the second step by using only 1.8 wt % TREN per RP (Table 2; entry 3b).

**Table 2:** Reaction Conditions and Properties of RPs Recovered from PUFs by Two-Step Aminolysis^a^

		amino/urethane group ratio in equivalents									
entry	PUF type	TREN	HMDA	PUF/V/reagent (g/g/g)	reagent (wt %)	urethane group content (mol %)	amino end group content (mol %)	TDA content (wt %)	allyl group	*M*_w_ (kg mol^–1^)	*Đ*
1a	PUF5611 I-step	2.25		6/3/0.44	7.3	5.8	5.8	6.0	no	3.1	1.02
1b	RP5611 II-step	4.00		6 RP/0.07 TREN	1.2^b^	0	0	7.7	no	3.0	1.02
2a	PUF4811 I-step	2.25		6/3/0.38	6.3	6.4	6.2	6.4	no	3.5	1.02
2b	RP4811 II-step	4.00		6 RP/0.07 TREN	1.1^b^	0	0	8.5	no	3.5	1.02
3a	PUF5611 I-step		4.00	6/3/0.92	15.3	7.3	6.9	7.1	no	3.1	1.02
3b	RP5611 II-step	6.00		6 RP/0.10 TREN	1.8^b^	0	0	9.6	no	3.0	1.02

a The contents of urethane groups, TDA, and amino end groups in RP were calculated according to eqs S1–S3, respectively.

b In the second aminolysis step, TREN is given in wt % per RP.

Purified PPO- and P(PO-*co*-EO)-based RPs obtained by the two-step aminolysis are close equivalents of the corresponding VPs in terms of structural and molar mass characteristics as well as purity, as shown by the results of the comprehensive characterization of RPs (Figures 3, S13, Tables S4, and S8). Therefore, they can be used as medium for PUF degradation instead of VP. The aminolysis of PUF5611 with 13.2 wt % TREN was carried out with RP medium and also in bulk without any medium (Table 1; entries 11 and 12 and Figure S9); however, in the latter case, two preheating cycles were required to partially degrade the cross-linked structure of PUF and allow efficient stirring of the reaction mixture in the main heating cycle. The quality of the recovered polyols and the amount of TDA formed in both cases are comparable to that of RP produced with the VP medium (Table 1; entry 4) when the dilution of RPs is taken into account in the calculation of TDA.

### Synthesis of Flexible PUFs from RPs

Purified PPO-based RPs containing 6.9 and 0 mol % residual urethane groups (Figure S10) were used to synthesize new flexible PUFs without adjusting the PUF formulation or synthesis conditions compared to PUF prepared from 100% VP. The PUF prepared from fully hydroxyl-functionalized RP exhibits an open-cell morphology, just like the PUF prepared from VP (Figure S11). In contrast, PUF prepared from partially aromatic amino-functionalized polyol shows a more closed-cell morphology and a slightly smaller average pore size.

Compression tests show comparable mechanical properties (compressive moduli and stress at 40% compression) of PUFs made from VP and fully hydroxyl-functionalized RP, while PUFs made from partially aromatic amino-functionalized RP show higher compressive modulus and stress at 40% compression, which can be attributed to a more closed-cell morphology (Table S7 and Figure S12). Flexible PUFs are widely used cushioning materials, so good recovery after prolonged compression is desirable. Compression set property as a potential predictor of height and load bearing loss reflecting changes in the PUF network was measured at 50% strain at 70 °C for 22 h and determined according to eq S7. PUFs synthesized from VP and fully hydroxyl-functionalized RP have comparable compression set values, while PUF prepared from partially amino-functionalized RP has a higher value (Table S7), indicating slightly lower durability of this particular PUF, but still within specifications for standard flexible foams.^56^ These results suggest that the aromatic amino end groups, similar to the carboxyl end groups of the acidolysis-derived RP,^7^ influence the polymerization process, albeit to a much lesser extent. This is evident from the shorter gel time of PUF synthesized from partially amino-functionalized RP (Table S6), which is due to the higher reactivity of amino groups compared to hydroxyl groups.^54,55^

**Figure 4:**
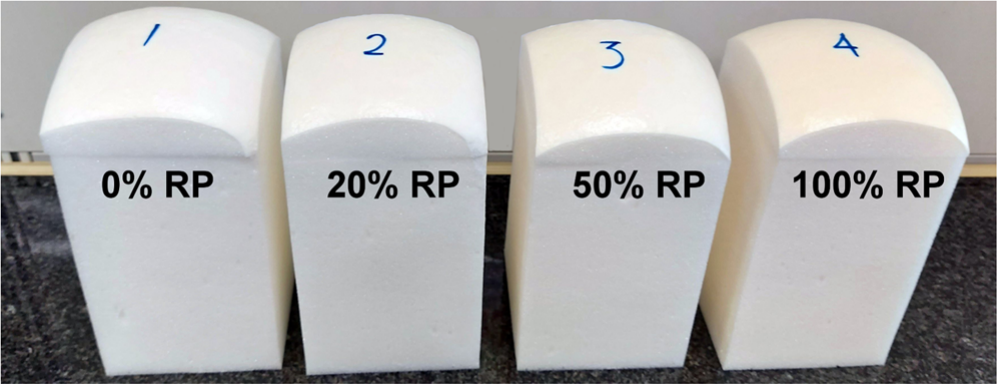
Photo of PUFs where different amounts of VP4811 in the PUF formulation were replaced with fully hydroxyl-functionalized RP4811: (1) 0%, (2) 20%, (3) 50%, and (4) 100%.

PUFs were also synthesized from more commonly used, fully hydroxyl-functionalized copolyether polyol-based RP (Figure 4 and Table S8) by replacing 0, 20, 50, and 100 wt % of VP in the PUF formulation with RP obtained by a two-step aminolysis procedure. The characteristic times (cream time, rise time) during the synthesis of copolyether polyol-based PUFs were insignificantly affected by the RP content in the PUF formulation (Table S10). The densities, porosities, and mechanical properties of all synthesized PUFs were highly comparable even when the PUF was synthesized exclusively from RP (Figure 4 and Table 3).

**Table 3:** Densities, Porosities, and Mechanical Properties of Copolyether Polyol-Based PUFs Synthesized with Different Amounts of RP_0% amino groups_ in the PUF Formulation

PUF number	density (kg m^–3^)	porosity (mm s^–1^)	average compression resistance 40% (kPa)	resilience (%)	compression set (%)	traction resistance (kPa)	elongation (%)
1	22.6	1.350	2.86	41	4.0	86	222
2	23.0	1.540	2.89	41	4.0	83	215
3	23.1	1.335	2.94	41	4.4	87	227
4	23.1	1.285	2.97	42	4.4	95	260

## Conclusions

MW-assisted aminolysis of flexible PUFs with reagents containing both primary and tertiary amino groups in the structure allows efficient degradation of the urethane groups in the PUF structure. Such reagents probably not only act as aminolysis agents but also prevent the recombination of isocyanate and hydroxyl groups formed during the thermal degradation of the PUFs back into the urethane bond by forming aliphatic urea, whereby the tertiary amino group of the reagent acts as a catalyst for this reaction. The amount of reagent that allows complete degradation of the urethane groups is much lower than that normally required for typical solvolysis reactions, especially when the aminolysis is carried out as a two-step process. At a degree of degradation of the urethane groups of 99% or more and after purification of the crude RPs, the yield of aminolysis-derived RPs is typically 83–86%. Since the RPs obtained by a two-step aminolysis process are structurally equivalent to the corresponding VPs, they can be used as a full substitute for VP in the PUF formulation for the synthesis of high-quality flexible PUFs whose morphology and mechanical properties are comparable to the PUFs synthesized from the corresponding VPs. For the same reason, RP can also be used as a medium in the aminolysis process instead of VP, which improves the sustainability of the process. Furthermore, the high quality of RPs is not affected when the PUFs from RPs are subjected to the aminolysis process again, showing that PUFs can be recycled at least twice with this process.

## Supporting Information Available

Experimental conditions and results of characterization of polyols by ^1^H NMR, HPLC, SEC/UV-MALS-RI, MALDI-TOF MS, FTIR, determination of OH number, acid value, and water content; experimental conditions for the synthesis of PUFs; and their characterization (PDF)

## Abbreviations

EO: ethylene oxide
Eqs: equivalents
EtOAc: ethyl acetate
DABCO: triethylenediamine
DMA: dynamic mechanical analyzer
EtOAc: ethyl acetate
FT-IR: Fourier transform infrared spectroscopy
HMDA: hexamethylenediamine
MALDI-TOF MS: matrix-assisted laser desorption/ionization time-of-flight mass spectrometry
MDA: methylene diphenyl diamine
MDI: methylene diphenyl diisocyanate
MW: microwave
NMR: nuclear magnetic resonance
PEI: poly(ethylenimine)
PO: propylene oxide
PPO: poly(propylene oxide)
PUF: polyurethane foam
RP: recovered polyol
SEC/UV-MALS-RI: size exclusion chromatography coupled with ultraviolet, multiangle light scattering, and refractive index detectors
TDA: toluene diamine
TDI: toluene diisocyanate
TREN: tris(2-aminoethyl)amine
VP: virgin polyol
