# Supplementary material for: Chemical Recycling of Flexible Polyurethane Foams by Aminolysis to Recover High-Quality Polyols
Source: ACS Sustain Chem Eng. 2023 Jul 10;11(29):10864–73. doi: 10.1021/acssuschemeng.3c02311 (PMC10369675; doi:10.1021/acssuschemeng.3c02311)
Supplement: Supplementary file 1 — sc3c02311_si_001.pdf [file sc3c02311_si_001.pdf]

Supporting information

Chemical Recycling of Flexible Polyurethane Foams by Aminolysis to Recover High Quality Polyols

Maja Grdadolnik[†], *Blaž Zdovc*[†], *Ana Drinčić*[†], *Ozgun Can Onder*[†], *Petra Utroša*[†], *Susana Garcia Ramos*[‡], *Enrique Dominguez Ramos*[‡], *David Pahovnik*[†], *and Ema Žagar*[†,*]

[†] Department of Polymer Chemistry and Technology, National Institute of Chemistry, Hajdrihova 19, Ljubljana, SI-1000, Slovenia

[‡] Intermediates Technical Service & Development department, Repsol Quimica S.A, Mendez Álvaro 44 CP28045, Madrid, Spain.

* Corresponding Author: Ema Žagar, E-mail: ema.zagar@ki.si, Phone: +386 1 4760 203

Number of pages: 23

Number of figures: 13

Number of tables: 10

Table of Contents

Supporting information S1

List of Tables S2

List of Figures S3

Experimental S5

Materials S5

Synthesis of flexible PUFs S6

Characterization methods for polyols S6

Nuclear magnetic resonance (NMR) S6

Matrix-assisted laser desorption / ionization time-of-flight mass spectrometry (MALDI-TOF MS) S6

Size-exclusion chromatography coupled with a multidetection system consisting of a UV-detector, a multi-angle light scattering photometer (MALS), and a refractive index (RI) detector (SEC/UV-MALS-RI). S7

Liquid adsorption chromatography for separation of RPs according to functionality S7

Fourier-transform infrared (FTIR) spectra of RPs S7

Acid value (AV) S7

Hydroxyl number (OH_{number}) S7

The water content S8

Characterization of flexible PUFs S8

Homopolyether polyol-based PUFs S8

Copolyether polyol-based PUF S8

Results S9

Results of characterization of RPs and VPs S9

Characteristics of homopolyether polyols used for the synthesis of flexible PUFs S19

Characteristics of copolyether polyols used for the synthesis of flexible PUFs S22

Flexible PUFs synthesized from copolyether polyols S23

List of Tables

Table S1. Signal assignment in ^{1}H NMR spectra of RPs. S9

Table S2. Molar mass characteristics (weight-average molar mass, M_w, and dispersity, $Đ = M_w/M_n$) of RPs and corresponding ALCUPOL® F-5611 virgin polyol (VP5611) as determined by SEC/MALS-RI together with residual urethane group content, allyl group content, and TDA content in RPs as determined by ^{1}H NMR spectroscopy. RPs were recovered from PUF5611 at various reaction temperatures and times using 1.25 Eqs of TREN amino per PUF urethane group. S10

Table S3. Reaction conditions and properties of RPs recovered from PUFs by aminolysis with PEI-600 and PEI-1800 at 220 °C after 30 min. S14

Table S4. Properties of VP5611 and corresponding RPs recovered from PPO-based PUF by one- and two-step aminolysis with TREN. S20

Table S5. Formulations used for PUF5611 synthesis. The amounts of the formulation components are given in “parts per hundred polyol” (pphp; w/w). The amount of PPO-based polyol used for a single PUF synthesis was 16 g. S20

Table S6. Characteristic times during synthesis of PPO-based PUFs. S20

Table S7. Mechanical properties of PPO-based PUFs. S21

Table S8. Properties of VP4811 and corresponding $RP_{0\%\ amino\ groups}$ recovered from P(PO-*co*-EO)-based PUF4811 by two-step aminolysis with TREN S23

Table S9. Formulations used for PUF4811 synthesis. The amounts of the formulation components are given in “parts per hundred polyol” (pphp; w/w). The amount of polyol used for a single PUF synthesis was 100 g. S23

Table S10. Characteristic times during synthesis of P(PO-*co*-EO)-based PUFs. S23

List of Figures

Figure S1. (a) Magnified and (b) full SEC/UV-RI chromatograms of crude RPs obtained by aminolysis of PPO-based PUF with TREN at 210 °C after reaction times 15, 30 and 40 min and at reaction temperatures of 210, 220 and 230 °C after 30 min. The solid and dashed curves represent the RI and UV detector responses, respectively, while the dotted lines show the molar mass as a function of elution volume. S11

Figure S2. ^{1}H NMR spectra of crude RPs obtained by aminolysis of PPO-based PUF with different amounts of TREN at 220 °C after 30 min. The ^{1}H NMR spectra were normalized to the signal of methyl group (marked with a) of the PO repeating units. S12

Figure S3. Top: Crude reaction mixtures before (a, b, c) and after (d, e, f) centrifugation at 9000 rpm obtained by one-step aminolysis of PPO-based PUF at 220 °C for 30 min with different amounts of TREN. Photos of the hard segments and crude recycled polyol isolated by pouring from the centrifuged reaction mixture. The crude polyol was further purified by liquid-liquid extraction to obtain purified RP. Below is ^{1}H NMR spectrum of the hard segments recorded in DMSO-d_6 (residual DMSO marked with *). The spectrum also shows the presence of residual polyol adsorbed on the hard segments and TDA isomers (signals marked with ▽). S13

Figure S4. Magnified regions of the ^{1}H NMR spectra of VP5611 and the corresponding purified RPs recovered from PPO-based PUF by one-step aminolysis at 220 °C for 30 min with TREN and HMDA with and without DABCO catalyst. The ^{1}H NMR spectra were normalized to the methyl group of the PO repeating units. S14

Figure S5. Magnified (a) ^{1}H NMR spectra, (b) SEC/UV-RI chromatograms and (c) FTIR spectra of VP5611 and corresponding purified RPs recovered from PPO-based PUF by aminolysis at 220 °C after 30 min with 11.0 and 14.2 wt % PEI-600 and 14.8 wt % PEI-1800. The ^{1}H NMR spectra were normalized to the signal of methyl group of the PO repeating units. The solid and dashed curves in SEC/UV-RI chromatograms represent the RI and UV detector responses, respectively, while the dotted lines show the molar mass as a function of elution volume. S15

Figure S6. Magnified (a) ^{1}H NMR spectra and (b) SEC/UV-RI chromatograms of VP4811 and corresponding purified RPs recovered from P(PO-*co*-EO)-based post-industrial and post-consumer PUF4811 by aminolysis at 220 °C after 30 min with TREN and PEI-600. The solid and dashed curves in the SEC/UV-RI chromatograms represent the RI and UV detector responses, respectively, while the dotted lines show the molar mass as a function of elution volume. Below is a photo of post-consumer PUF waste. S16

Figure S7. Magnified (a) ^{1}H NMR spectra and (b) SEC/UV-RI chromatograms of VP2832 and corresponding P(PO-*co*-EO)-based RP recovered from MDI-based post-industrial high resilience PUF by aminolysis at 220 °C for 30 min with 9.3 wt % TREN. The solid and dashed curves in the SEC/UV-RI chromatograms represent the RI and UV detector responses, respectively, while the dotted lines show the molar mass as a function of elution volume. S17

Figure S8. (a) Magnified ^{1}H NMR spectra and (b) SEC/UV-RI chromatograms of VP5611 and the corresponding twice-recycled polyols recovered from PPO-based PUF by aminolysis at 220 °C after 30 min with 13.2 wt % TREN and 13.2 wt % PEI-600. The solid and dashed curves in the SEC/UV-RI chromatograms represent the RI and UV detector responses, respectively, while the dotted lines show the molar mass as a function of elution volume. S17

Figure S9. (a) MALDI-TOF mass spectra, (b) magnified ^{1}H NMR spectra, (c) SEC/UV-RI chromatograms and d) FTIR spectra of VP5611 and the corresponding purified RPs obtained with 13.2 wt % TREN using RP as a medium and without the use of any medium (bulk) at 220 °C after 30 min. The ^{1}H NMR spectra were normalized to the signal of methyl group of the PO repeating units. The solid and dashed curves in the SEC/UV-RI chromatograms represent the RI and UV detector responses, respectively. S18

Figure S10. Magnified (a) ^{1}H NMR spectra, (b) FTIR spectra, (c) SEC/UV-RI chromatograms and (d) MALDI-TOF mass spectra of VP5611 and the corresponding RPs containing 6.9 mol % and 0.0 mol % aromatic amino end groups, recovered from PPO-based PUF by one- and two-step aminolysis with TREN, respectively. The solid and dashed curves in the SEC/UV-RI chromatograms represent the RI and UV detector responses, respectively, while the dotted lines show the molar mass as a function of elution volume. S19

Figure S11. Photos and SEM cross-sectional images of PUFs prepared according to the formulation in Table S5 with (a) 100% VP5611, (b) 100% $RP_{0\%\ amino\ groups}$ (obtained by two-step aminolysis with TREN), (c) 100% $RP_{6.9\%\ amino\ groups}$ (obtained by one-step aminolysis with TREN). Average pore sizes in µm are given in the lower left corners of the SEM images..S21

Figure S12. Compressive stress-strain curves of PPO-based PUFs prepared from 100% VP5611, 100% $RP_{0\%\ amino\ groups}$ (obtained by two-step aminolysis with TREN) and 100% $RP_{6.9\%\ amino\ groups}$ (obtained by one-step aminolysis with TREN). S22

Figure S13. (a) ^{1}H NMR spectra, (b) SEC/UV-RI chromatograms and (c) FTIR spectra of fully hydroxyl-functionalized copolyether-based RP obtained by two-step aminolysis of P(PO-*co*-EO)-based PUF4811 with TREN and the corresponding virgin polyol of the same type (VP4811). The ^{1}H NMR spectra were normalized to the signal methyl group of the PO repeating units. The solid and dashed curves in the SEC/UV-RI chromatograms represent the RI and UV detector responses, respectively, while the dotted lines show the molar mass as a function of elution volume. S22

Experimental

Materials

The virgin polyether polyols ALCUPOL® F-5611, ALCUPOL® F-4811 and ALCUPOL® F-2832 denoted as VP5611, VP4811 and VP2832, respectively, and the PUFs synthesized therefrom were supplied by Repsol S.A.. VP5611 is a trifunctional propylene oxide (PO) based homopolyether polyol with a hydroxyl number of 56 mg KOH g^{-1} and a molar mass of 3.0 kg mol^{-1}. It contains a mixture of isomers (C7-9-alkyl 3-(3,5-di-*tert*-butyl-4-hydroxyphenyl)propionate; CAS 125643-61-0) as a phenolic antioxidant and reaction products of *N*-phenylbenzenamine with 2,4,4-trimethylpentene; CAS 68411-46-1) as an amine antioxidant. VP4811 is a trifunctional copolyether polyol consisting of PO and ethylene oxide (EO) repeating units, with an EO content of 12 mol %. It has a hydroxyl number of 48 mg KOH g^{-1}, a molar mass of 3.5 kg mol^{-1} and it contains the same phenolic and amine antioxidants as VP5611. VP2832 is a trifunctional copolyether polyol consisting of PO and EO repeating units, with an EO content of 15.2 mol %. It has a hydroxyl number of 28 mg KOH g^{-1} and a molar mass of 6.0 kg mol^{-1}. PUF5611 and PUF4811 were synthesized from the corresponding F-5611 and F-4811 ALCUPOL® polyols (polyol content: 66.1 and 65.9 wt %, respectively), an isomer mixture of 2,4- and 2,6-toluene diisocyanate (TDI) with isomer ratio of 80/20 (TDI index of 107), and water as a foaming agent to chemically produce CO_2. Kosmos® 29 (tin(II) octoate), TEGOAMIN® 33 and TEGOAMIN® BDE were used as gelling and foaming catalysts, while silicone TEGOSTAB® BF 2370 was used as a surfactant to control cell size and opening. Post-industrial and post-consumer PUFs were supplied by Repsol S.A. and are all based on copolyether polyol and TDI. PUF2832 was synthesized from the corresponding F-2832 ALCUPOL® polyol (polyol content: 61.7 wt %), methylene diphenyl diisocyanate (MDI) and water as a foaming agent. DABCO® NE300, dibutyltin dilaurateoctoate (DBTDL), TEGOAMIN® 33 and TEGOAMIN® BDE were used as gelling and foaming catalysts, while silicone TEGOSTAB® B 8747 LF2 was used as a surfactant.

Tris(2-aminoethyl)amine (TREN; Sigma-Aldrich, Germany), polyethylenimine with number-average molar mass of 600 and 1800 g mol^{-1} (PEI-600 and PEI-1800; Sigma-Aldrich, Germany), hexamethylenediamine (HMDA; Sigma-Aldrich, Germany) were used as aminolysis reagents and triethylenediamine (DABCO; Aldrich, Germany) as a catalyst. Acetonitrile (ACN; ≥99.9%; Riedel de Haën, Germany), methanol (MeOH; ≥99.9%; Merck, Germany), formic acid (FA; ≥98%; Fluka, Germany), ammonium formate (AmFm; ≥97%; Fluka, Germany), Milli-Q water (MQ) with 18.2 MΩ resistivity and polyethylene glycol (PEG) with weight-average molar mass of 4.0 kg mol^{-1} were used to perform HPLC and size-exclusion chromatographic (SEC) experiments. Deuterated dimethyl sulfoxide (DMSO-d_6; Eurisotop, Germany) and trifluoroacetic acid (TFA; 99%; Aldrich, Germany) were used for ^{1}H NMR experiments. Hydrochloric acid (HCl; Eurisotop, Germany), EtOAc (Honeywell, USA) and MQ were used for purification of RPs. Imidazole (Sigma-Aldrich, Germany), pyridine (Sigma-Aldrich, Germany), phenolphthalein (Merck, Germany), phthalic anhydride (Merck, Germany), potassium hydrogen phthalate (KHP; Acros Organics, USA), sodium hydroxide (NaOH; Honeywell, USA), and ethyl alcohol (EtOH; Carlo Erba, Italy) were used to determine hydroxyl number and acid value of RPs. Aquastar water standard 0.01% (Supelco, Germany), Aquastar CombiCoulomat reagent (Supelco, Germany) and chloroform ($CHCl_3$; Honeywell, Fluka) were used to determine water content in RPs. Tetrahydrofuran (THF; p.a.), 2,5-dihydroxybenzoic acid (≥99.0%), sodium trifluoroacetate (≥98.0%) and poly(methyl methacrylate) standards (PMMA; MALDI validation set, Fluka Analytical) were used to perform MALDI TOF MS experiments. TDI Ongronate (TDI 80/20), Kosmos® 29 (tin(II) octoate catalyst), B11 tertiary amine catalyst and silicone 2370 were used for flexible homopolyether PUF synthesis and were obtained from Plama-pur, Slovenia. TDI 80/20, Kosmos® 29 (tin(II) octoate catalyst), TEGOAMIN® 33, TEGOAMIN® BDE and silicone L-620 LV were used for copolyether PUF synthesis by Repsol S.A. All chemicals were used as received.

Synthesis of flexible PUFs

The flexible PUFs were synthesized from PPO-based polyols or copolymeric polyols by the standard cup procedure according to the formulations in Tables S5 and S9, respectively. The homogeneous mixture consisting of polyol, water as blowing agent, silicone surfactant and catalysts was mixed together with TDI80/20 (TDI index of 107) for 5−10 s using Eurostar 40 digital mechanical stirrer (IKA, Germany). Then, the mixture was quickly poured into a cardboard cup (8.5 cm × 8.5 cm × 8.5 cm for PPO-based PUFs and 15 cm × 15 cm × 15 cm for copolymer-based PUFs). The foams were stored in a dark and dry place for 72 h to cure. The specimens were then cut for analysis of morphology and mechanical properties.

Characterization methods for polyols

Nuclear magnetic resonance (NMR). ^{1}H NMR spectra were recorded at room temperature in DMSO-d_6 with or without added TFA using a Bruker AVANCE NEO 600 MHz instrument (Bruker Corporation, USA). Chemical shifts (δ) are given in ppm relative to a DMSO-d_6 residual peak.

Content of residual urethane groups in PPO-based RPs was determined from ^{1}H NMR spectra recorded in DMSO-d_6 with added TFA to shift the signal of the amino groups, which overlaps with the signal of the polyol methyne groups adjacent to the urethane groups, to higher chemical shifts. The content of residual urethane groups in the RPs was determined according to eq S1 from the signal intensities of the polyol methyne protons (–NHCOO-C**H**<) adjacent to the residual urethane groups at δ 4.88 ppm (c') and the protons of the polyol methyl group (–C**H**$_3$) at δ 1.04 ppm (a) , assuming 50 PO repeat units in the polyol arms and polyol functionality of 3.

$$\textbf{\textit{Urethane group (–NHCOO–) content}}\ \textbf{(mol \%)} = \frac{I(-\mathrm{C}H<)_{\mathrm{c'}} \times 50}{I(-\mathrm{C}H_3)_{\mathrm{PO}}} \times 100 \qquad \text{(S1)}$$

The TDA content in crude PPO-based RPs was determined from the ^{1}H NMR spectra recorded in DMSO-d_6 according to eq S2 from the signal intensities of the methyl groups of the TDA isomers (δ 1.79 for 2,6-TDA and 1.88 ppm for 2,4-TDA; signals e and h, respectively) and the methyl group (a) of the PO repeating unit of the polyol at δ 1.04 ppm.

$$\textbf{\textit{TDA content}}\ \textbf{(wt \%)} = \frac{I(-\mathrm{C}H_3)_{\mathrm{TDA}} \times \mathrm{M(TDA)}}{\left(I(-\mathrm{C}H_3)_{\mathrm{PO}} \times \mathrm{M(PO)} + I(-\mathrm{C}H_3)_{\mathrm{TDA}} \times \mathrm{M(TDA)}\right)} \times 100 \qquad \text{(S2)}$$

The content of amino (–NH$_2$) end groups in PPO-based RPs was determined from ^{1}H NMR spectra of the purified RPs recorded in DMSO-d_6 according to eq S3 from the signal intensities of the methyl groups of the TDA isomer moieties attached to the polyol via the urethane groups (δ 2.00, 1.96 and 1.87 ppm for both isomers; denoted as d) and the methyl group (a) of the PO repeating unit of the polyol at δ 1.04 ppm, assuming 50 PO repeat units in the polyol arms and polyol functionality of 3.

$$\textbf{\textit{–NH}}_{\mathbf{2}}\ \textbf{\textit{content}}\ \textbf{(mol \%)} = \frac{I(-\mathrm{C}H_3)_{\mathrm{d}} \times 50}{I(-\mathrm{C}H_3)_{\mathrm{PO}} \times 3} \times 100 \qquad \text{(S3)}$$

Matrix-assisted laser desorption / ionization time-of-flight mass spectrometry (MALDI-TOF MS) measurements were performed using a Bruker UltrafleXtreme MALDI-TOF mass spectrometer (Bruker Daltonics, Germany). Polyol samples were dissolved in THF (c = 10 mg mL^{-1}) and mixed with a solution of the matrix, 2,5-dihydroxybenzoic acid in THF (c = 30 mg mL^{-1}) and sodium trifluoroacetate in THF (c = 10 mg mL^{-1}), in a volume ratio of 1/10/3. A 0.4 μL of the prepared solution was spotted onto the target plate (dried-droplet method). The mass spectra of the samples were recorded in reflective positive ion mode. Calibration was performed externally using a mixture

of PMMA standards dissolved in THF covering the measured molecular weight range. Sample preparation for the standard mixture was the same as for the samples. The standard mixture was spotted to the nearest neighbour positions.

Size-exclusion chromatography coupled with a multidetection system consisting of a UV-detector, a multi-angle light scattering photometer (MALS), and a refractive index (RI) detector (SEC/UV-MALS-RI). The molar mass characteristics (M_w, M_n, dispersity: $Đ = M_w/M_n$) and purity of RPs were determined using SEC connected to a UV detector operating at a wavelength of 280 nm (Agilent Technologies, USA), a DAWN multi-angle light scattering photometer (Wyatt Technology Corp., USA), and an Optilab interferometric refractometer (RI) (Wyatt Technology Corp., USA). Separations were performed at room temperature using a TSKgel Alpha-2500 SEC column (7.8 mm ID × 30.0 cm L, particle size 7 µm, and exclusion limit 10 kDa) with a precolumn (Tosoh Bioscience GmbH, Germany). Methanol (MeOH) was used as the solvent and mobile phase at a flow rate of 0.7 mL min^{-1}. Toluene was used to calibrate the 90° LS detector, while the other detectors were normalized with the standard polyethylene glycol (PEG) with a weight-average molar mass of 4 kDa and a dispersity of 1.02. The typical RP concentration in MeOH was 1.0×10^{-2} g mL^{-1}, while the injection volume was typically 100 μL. The specific refractive index increment (dn/dc) required to calculate the molar masses of RPs was determined from corresponding virgin polyols assuming 100% mass recovery of the samples from the column. Astra 8 software was used for data acquisition and analysis (Wyatt Technology Corp., USA).

Liquid adsorption chromatography for separation of RPs according to functionality

Separation of RPs according to functionality. Liquid adsorption chromatography (LAC) experiments were performed on a SHARC 1 column (4.6 mm × 150 mm, 100 Å, 5 μm; SIELC Techonologies, USA) at a temperature of 25 °C using a thermostatted oven. An HPLC pump (Agilent 1260, Agilent Technologies, USA) provided delivery of the isocratic mobile phase consisting of 75% acetonitrile (ACN) with 3.00 vol % formic acid (FA) and 0.048 vol % MQ-water (MQ) and 25% MeOH with 0.1 mg mL^{-1} ammonium formate (AmFm), at a constant flow rate of 1 mL min^{-1}. For detection, a UV detector (VWD) operating at a wavelength of 283 nm and an evaporative light-scattering (ELS) detector 1260 Infinity (both Agilent Technologies, USA) were connected in series.

Fourier-transform infrared (FTIR) spectra of RPs were acquired in ATR mode with a spectral resolution of 4 cm^{-1} using a Spectrum One FTIR spectrometer (Perkin-Elmer, Waltham, USA).

Acid value (AV) was determined using the adapted standard ASTM D4662-08 (Reapproved 2011), where instead of a mixture of toluene and ethanol only ethanol was used. Approximately 1 g of polyol was dissolved in 5 mL of ethanol and a solution of phenolphthalein in ethanol was used as a color indicator. Titrations were performed in triplicate using 0.01 N NaOH solution to determine the average value. The end point of the titration was determined visually by a change in color of the solution to pink. The AV was calculated using eq S4:

$$\boldsymbol{AV} = \frac{(A-B) \times 56.1 \times N}{w} \tag{S4}$$

where A is the volume of NaOH solution (mL) required to titrate the sample; B is the volume of NaOH solution (mL) required to titrate the blank; N is the normality of the NaOH solution, and w is the weight of the sample (g).

Hydroxyl number (OH_{number}) was determined according to ASTM D4274-05, where the esterification process of the polyol with phthalic anhydride is catalyzed by imidazole. An esterification reagent (phthalic anhydride) was used as a blank and phenolphthalein dissolved in pyridine was used as the color indicator. Titrations were performed in triplicate with an aqueous 0.5 N NaOH solution to determine the average value. The end point of the titration was determined visually by a change in color of the solution to pink. The OH number was calculated according to eq S5:

$$\boldsymbol{OH_{number}} = \frac{(B-A) \times 56.1 \times N}{w} \quad \text{(S5)}$$

where A is the volume of NaOH solution (mL) required to titrate the sample; B is the volume of NaOH solution (mL) required to titrate the blank; N is the normality of the NaOH solution and w is the weight of the sample in g.

Finally, the OH number was corrected considering AV and calculated according to eq S6.

$$\textbf{Corrected } \boldsymbol{OH_{number}} = \frac{(B-A) \times 56.1 \times N}{w} + AV \quad \text{(S6)}$$

The water content in the polyols was determined by the adapted standard method ASTM D4672-12, in which additionaly to methanol chloroform was used as solvent to improve the solubility of the polyols. Karl Fischer titration was performed using a C10S Compact KF coulometer (Mettler Toledo, Columbus, USA). Titrations were performed in triplicate, where around 1 g of polyol sample was titrated at once.

Characterization of flexible PUFs

Homopolyether polyol-based PUFs

Scanning electron microscopy (SEM). The morphology of the PUFs was studied by scanning electron microscopy (SEM) on a high-resolution SEM Zeiss Ultra plus instrument (Carl Zeiss, Germany). The foams were cut with a razor blade perpendicular to the foam rise direction. The obtained cross-sections were coated with a Gatan PECS 682 (Gatan, USA) with a 10 nm thick gold layer to dissipate the charge during SEM analysis. Pore size analysis was performed on at least 100 pores in three different cross-sections using ImageJ software.

Compressive properties of PUFs were assessed by a DMA Q800 dynamic mechanical analyzer (TA Instruments) and 40 mm diameter compression discs. The specimens of a cuboid shape (10 mm height, 25 mm width, 25 mm length) were compressed at 50% min^{-1} up to 70% of the original specimen height and then decompressed at the same rate to the original specimen height. The procedure was repeated three times, and the stress-strain curve was recorded during the fourth compression cycle. From the stress-strain curve, the Young's modulus in the initial linear range, and the stress at 40% strain were determined. The average values of three specimens with standard errors are given for each sample.

Compression set was determined on the cuboid specimens (10 mm height, 25 mm width, 25 mm length) by compressing them to 50% of the initial height (d_0) and heating them to 70 °C. After 22 h, the foams were allowed to recover for 30 min at ambient conditions, and then the foam height (d_r) was measured. The compression set was calculated according to eq S7:

$$\textbf{Compression set (\%)} = \frac{d_0 - d_r}{d_0} \times 100 \quad \text{(S7)}$$

where d_0 and d_r are the heights of the original specimen and the specimen after compression testing, respectively.

Copolyether polyol-based PUF

Physical properties of copolyether polyol-based PUFs were determined according to ISO 845 for PUF density, ISO 3386 for compression resistance, ISO 8307 for resilience, ISO 1856 for compression set, ISO 9237 for porosity, ISO 1798 for tensile strength, and ISO 1798 for elongation.

Results

Results of characterization of RPs and VPs

Table S1. Signal assignment in ^{1}H NMR spectra of RPs.

Compound	Molecular structure	Characteristic chemical shifts (ppm)
PPO-polyol	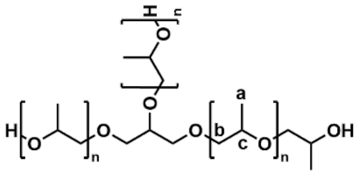	**a**: 1.04 ppm<br>**b**, **c**: 3.15–3.70 ppm<br>-O**H**: 4.40 ppm
Amino-functionalized PPO-polyol	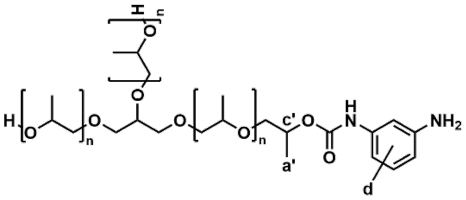	**a'**: 1.18 ppm<br>**d**: 1.87, 1.96, 2.00 ppm<br>**c'**: 4.88 ppm (DMSO-d_6 + TFA)<br>-N**H**COO-: 9.18, 8.58 and 8.47 ppm<br>-NH_2: 4.73, 4.78 and 4.80 ppm<br>H_{Ar}: 6.45, 6.54, 6.74 and 6.79 ppm<br>-O**H**; 4.40 ppm
Olefin-functionalized PPO-polyol	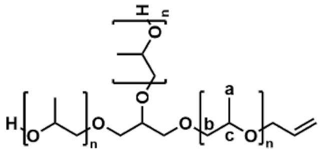	**a**: 1.04 ppm<br>**b**, **c**: 3.15–3.70 ppm<br>-O**H**: 4.40 ppm<br>=CH_2: 5.08 and 5.22 ppm
Dimer	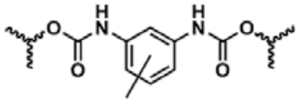	-CH_3: 2.10 and 2.12 ppm
2,6-toluene diamine (2,6-TDA)	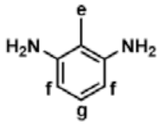	**e**: 1.79 ppm<br>**f**: 5.92 ppm<br>**g**: 6.54 ppm<br>-NH_2: 4.46 ppm
2,4-toluene diamine (2,4-TDA)	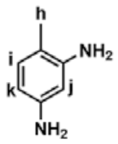	**h**: 1.88 ppm<br>**i**: 6.54 ppm<br>**j**: 5.88 ppm<br>**k**: 5.75 ppm<br>-NH_2: 4.43 and 4.44 ppm
Amino- or/and TREN-terminated (oligo)urea	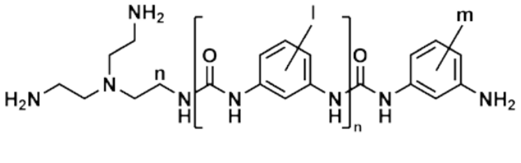	**l**: 2.17 and 2.20 ppm<br>**m**: 1.94, 1.97–2.09 ppm<br>**n;** 2.90–3.30 ppm<br>Ar-NH_2: 4.68, 4.75 and 4.77 ppm<br>Ar-N**H**CON**H**-Ar: 8.58, 8.44, 8.12, 7.65, 7.52 and 7.44 ppm

Tris(2-aminetil)amine (TREN)	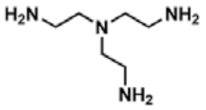	>N-C**H**$_2$-CH$_2$-NH$_2$: 2.36 ppm<br>>N-CH$_2$-C**H**$_2$-NH$_2$: 2.54 ppm
Polyethylenimine (PEI)	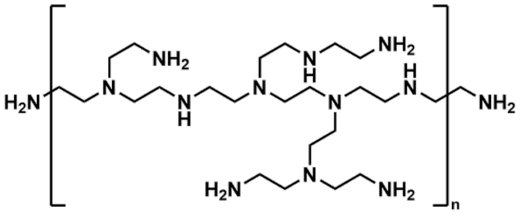	-C**H**$_2$-: 2.30–2.65 ppm
Hexamethylenediamine (HMDA)	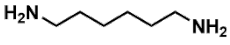	-C**H**$_2$-, γ: 1.25 ppm<br>-C**H**$_2$-, β: 1.32 ppm<br>-C**H**$_2$-NH$_2$: 2.50 ppm

Table S2. Molar mass characteristics (weight-average molar mass, M_w, and dispersity, $Đ = M_w/M_n$) of RPs and corresponding ALCUPOL® F-5611 virgin polyol (VP5611) as determined by SEC/MALS-RI together with residual urethane group content, allyl group content, and TDA content in RPs as determined by ^{1}H NMR spectroscopy. RPs were recovered from PUF5611 at various reaction temperatures and times using 1.25 Eqs of TREN amino per PUF urethane group.

Entry	*T* (°C)	*t* (min)	M_w (kg mol^{-1})	*Đ*	Residual urethane group content (mol %)	TDA content (wt %)	Allyl group content[a] (mol %)
VP5611	/	/	3.0	1.02	/	/	/
S1	210	15	3.5	1.07	19.0	2.2	0.3
S2	210	30	3.2	1.05	11.3	3.8	0.5
S3	210	40	3.1	1.02	10.0	3.9	0.5
S4	220	15	3.3	1.04	16.3	2.3	0.3
S5	220	30	3.1	1.03	10.8	3.7	0.6
S6	220	40	3.0	1.02	9.0	4.3	0.6
S7	230	15	3.2	1.04	10.0	3.4	0.8
S8	230	30	3.0	1.02	8.3	4.3	0.8
S9	230	40	3.0	1.02	8.0	5.4	0.8

[a] The content of allyl (C**H**$_2$-CH-R) end groups in the RPs was determined from ^{1}H NMR spectra according to eq S8 from the intensity of the methylene signal next to the double bond at 5.08 ppm and 5.22 ppm and the intensity of the methyl signal (–C**H**$_3$) of the polyol at δ 1.04 ppm, assuming that 50 PO repeat units are present in the polyol with a functionality of 3.

$$\textbf{\textit{Allyl group content}}\ \textbf{(mol \%)} = \frac{I(=CH_2) \times 50}{I(-CH_3)_{PO} \times 2} \times 100 \qquad \text{(S8)}$$

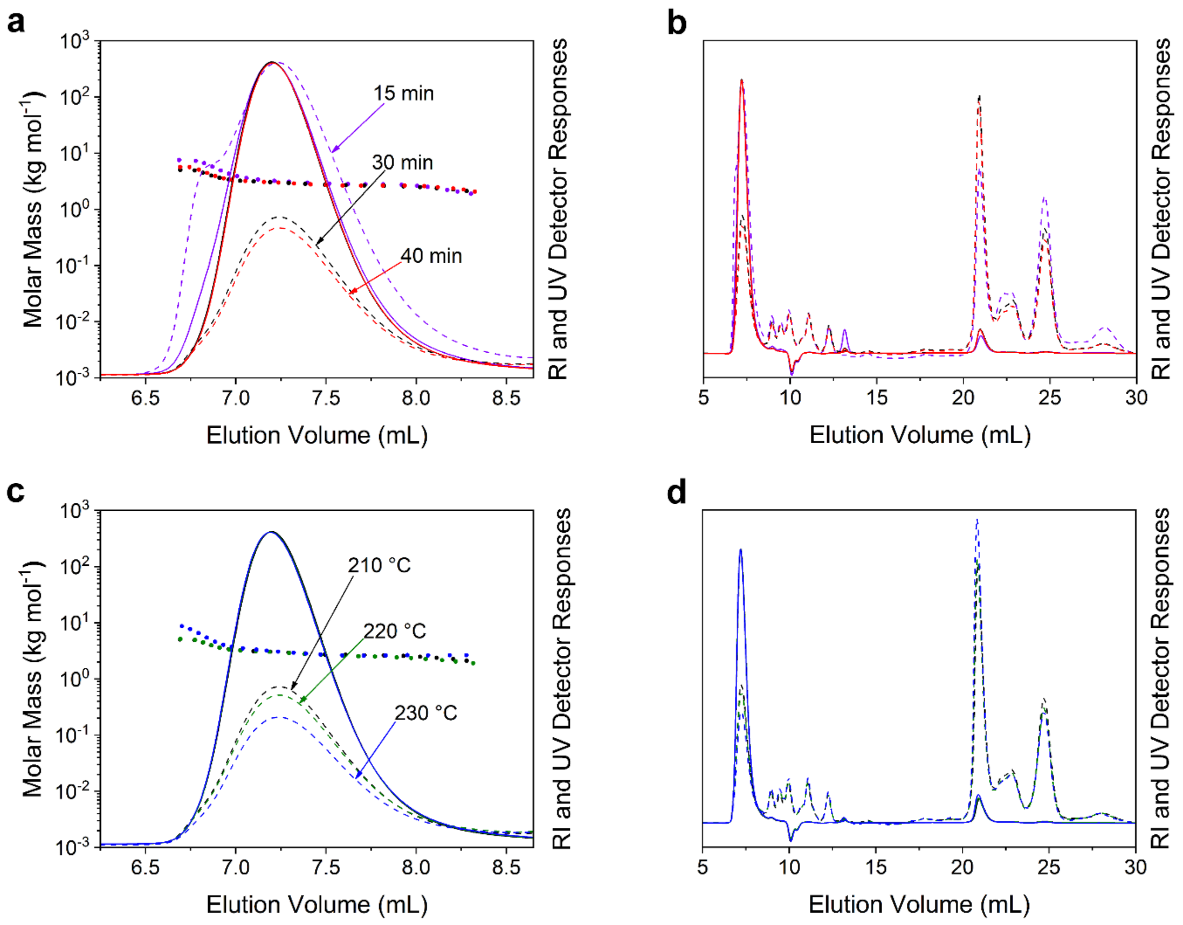

Figure S1. (a, c) Magnified and (b, d) full SEC/UV-RI chromatograms of crude RPs obtained by aminolysis of PPO-based PUF with TREN at 210 °C after reaction times 15, 30 and 40 min and at reaction temperatures of 210, 220 and 230 °C after 30 min. The solid and dashed curves represent the RI and UV detector responses, respectively, while the dotted lines show the molar mass as a function of elution volume.

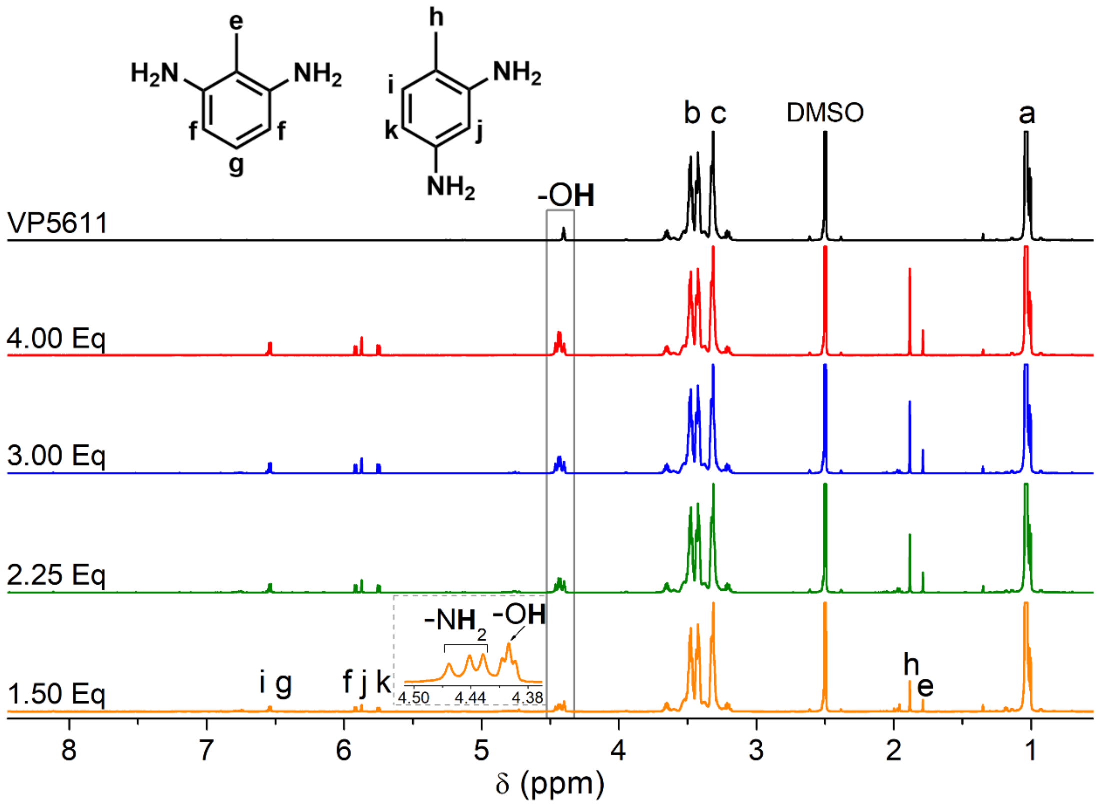

Figure S2. ^{1}H NMR spectra of crude RPs obtained by aminolysis of PPO-based PUF with different amounts of TREN at 220 °C after 30 min. The ^{1}H NMR spectra were normalized to the signal of methyl group (marked with a) of the PO repeating units.

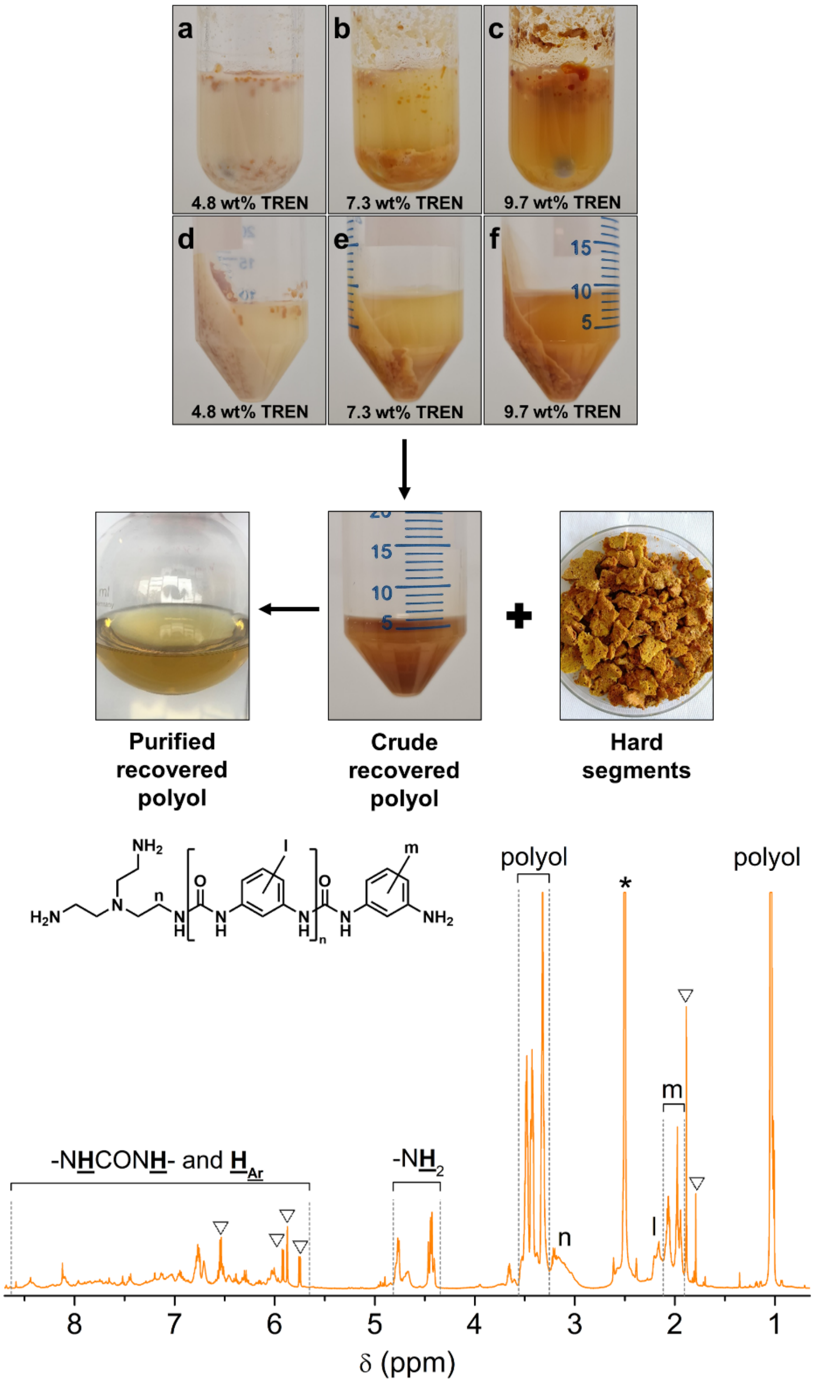

Figure S3. Top: Crude reaction mixtures before (a, b, c) and after (d, e, f) centrifugation at 9000 rpm obtained by one-step aminolysis of PPO-based PUF at 220 °C for 30 min with different amounts of TREN. Photos of the hard segments and crude recycled polyol isolated by pouring from the centrifuged reaction mixture. The crude polyol was further purified by liquid-liquid extraction to obtain purified RP. Below is ^{1}H NMR spectrum of the hard segments recorded in DMSO-d_6 (residual DMSO marked with *). The spectrum also shows the presence of residual polyol adsorbed on the hard segments and TDA isomers (signals marked with ▽).

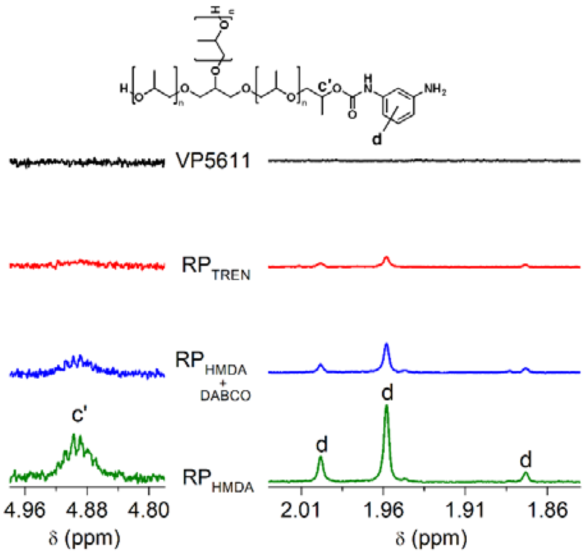

Figure S4. Magnified regions of the ^{1}H NMR spectra of VP5611 and the corresponding purified RPs recovered from PPO-based PUF by one-step aminolysis at 220 °C for 30 min with TREN and HMDA with and without DABCO catalyst. The ^{1}H NMR spectra were normalized to the methyl group of the PO repeating units.

Table S3. Reaction conditions and properties of RPs recovered from PUFs by aminolysis with PEI-600 and PEI-1800 at 220 °C after 30 min.

Entry	PUF type	Amino/Urethane group ratio in Eqs. PEI-600	PEI-1800	PUF/VP/Reagent (g/g/g)	Reagent /PUF (wt %)	Urethane group content (mol %)	Amino end group content(mol %)	TDA content (wt %)	Allyl group	M_w (kg mol^{-1})	*Đ*	*Yield* (%)
**1**	PUF5611	3.00	/	6/3/0.66	11.0	1.1	1.1	8.7	Yes	3.0	1.03	83
**2**	PUF5611	3.80	/	6/3/0.85	14.2	1.0	0.9	8.8	No	3.0	1.02	**
**3**	PUF5611	/	3.70	6/3/0.89	14.8	2.3	1.9	7.5	Yes	3.0	1.02	**
**4**[a]	PUF4811	3.80	/	6/3/0.74	12.3	1.3	1.3	10.3	No	3.5	1.02	82
**5**[a]	Post-consumer PUF	3.80	/	6/3/0.74	12.3	*	0.7	8.7	No	3.5	1.02	84
**6**	PUF5611 from RP	3.60	/	6/3/0. 79	13.2	*	1.0	8.4	No	3.0	1.02	86

Molar mass characteristics of VPs as determined by SEC/MALS-RI are M_w = 3.0 kg mol^{-1}, Đ = 1.02 for VP5611 and M_w = 3.5 kg mol^{-1}, Đ = 1.02 for VP4811.
The contents of urethane groups, TDA, and amino end groups in RP were calculated according to eqs S1, S2 and S3, respectively.
[a] The molar mass and the chemical composition of the P(PO-*co*-EO) polyol VP4811 were considered in the calculations of the contents of urethane and amino group, and TDA.
[*] The intensity of the polyol ^{1}H NMR methyne signal near the urethane group is too low for accurate quantification.
[**] Not determined.

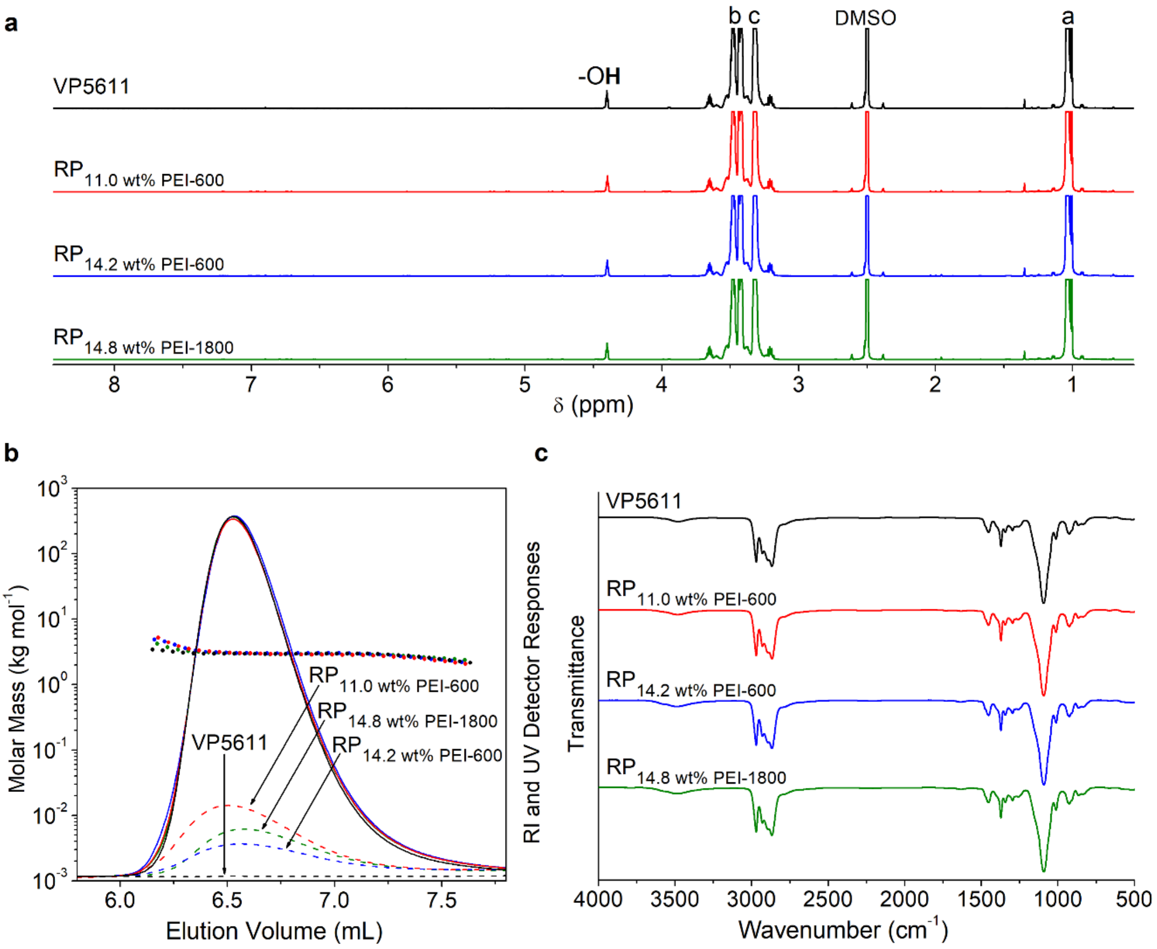

Figure S5. Magnified (a) ^{1}H NMR spectra, (b) SEC/UV-RI chromatograms and (c) FTIR spectra of VP5611 and corresponding purified RPs recovered from PPO-based PUF by aminolysis at 220 °C after 30 min with 11.0 and 14.2 wt % PEI-600 and 14.8 wt % PEI-1800. The ^{1}H NMR spectra were normalized to the signal of methyl group of the PO repeating units. The solid and dashed curves in SEC/UV-RI chromatograms represent the RI and UV detector responses, respectively, while the dotted lines show the molar mass as a function of elution volume.

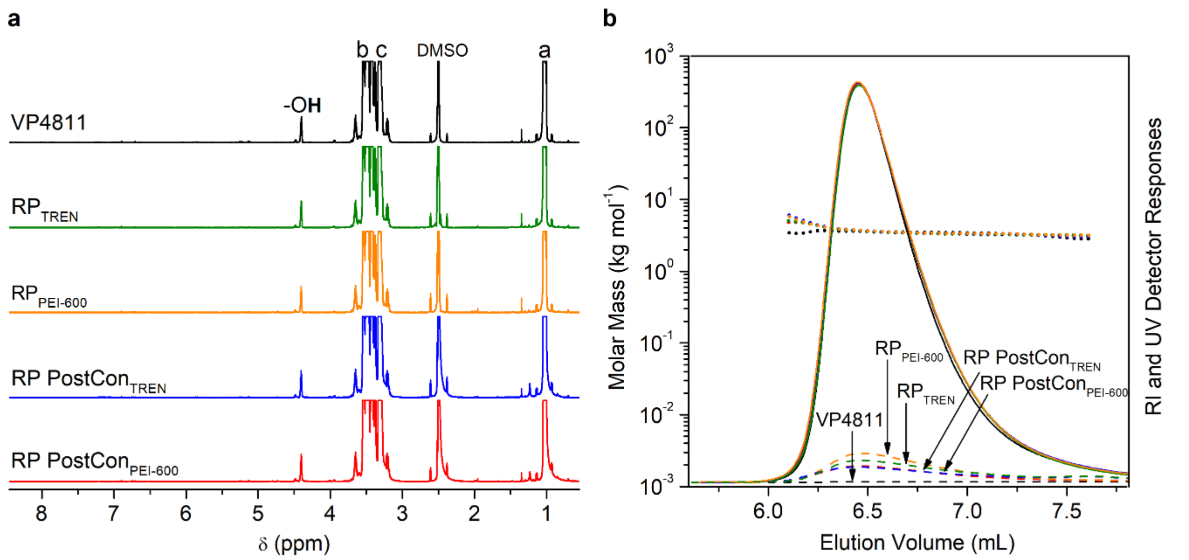

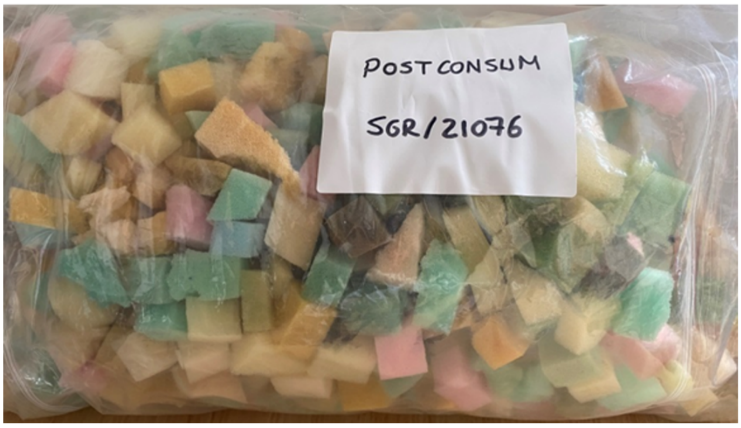

Figure S6. Magnified (a) ^{1}H NMR spectra and (b) SEC/UV-RI chromatograms of VP4811 and corresponding purified RPs recovered from P(PO-*co*-EO)-based post-industrial and post-consumer PUF4811 by aminolysis at 220 °C after 30 min with TREN and PEI-600. The solid and dashed curves in the SEC/UV-RI chromatograms represent the RI and UV detector responses, respectively, while the dotted lines show the molar mass as a function of elution volume. Below is a photo of post-consumer PUF waste.

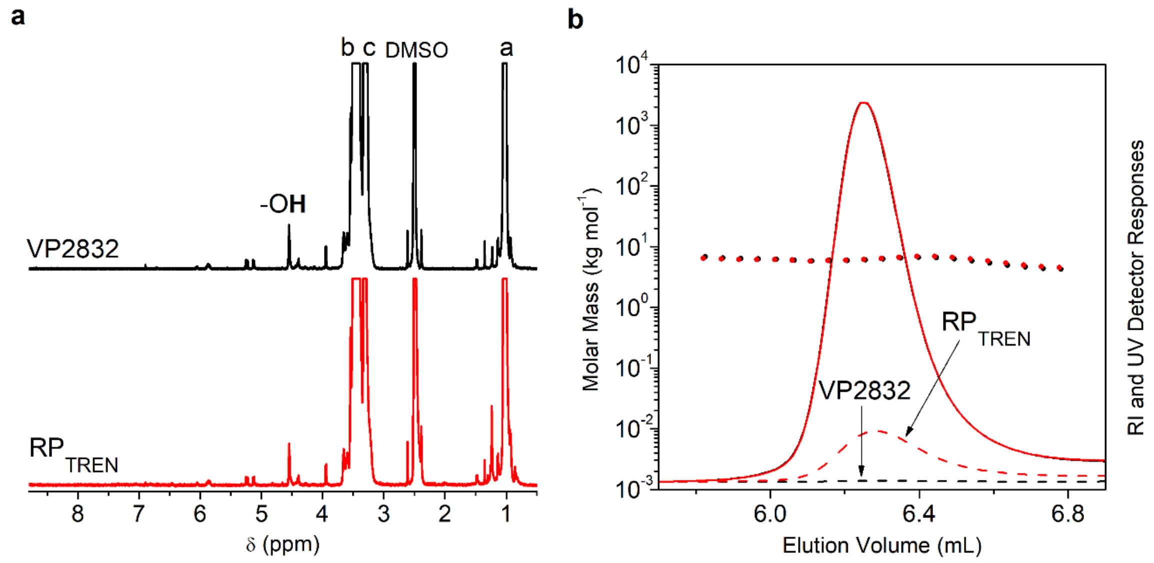

Figure S7. Magnified (a) ^{1}H NMR spectra and (b) SEC/UV-RI chromatograms of VP2832 and corresponding P(PO-*co*-EO)-based RP recovered from MDI-based post-industrial high resilience PUF by aminolysis at 220 °C for 30 min with 9.3 wt % TREN. The solid and dashed curves in the SEC/UV-RI chromatograms represent the RI and UV detector responses, respectively, while the dotted lines show the molar mass as a function of elution volume.

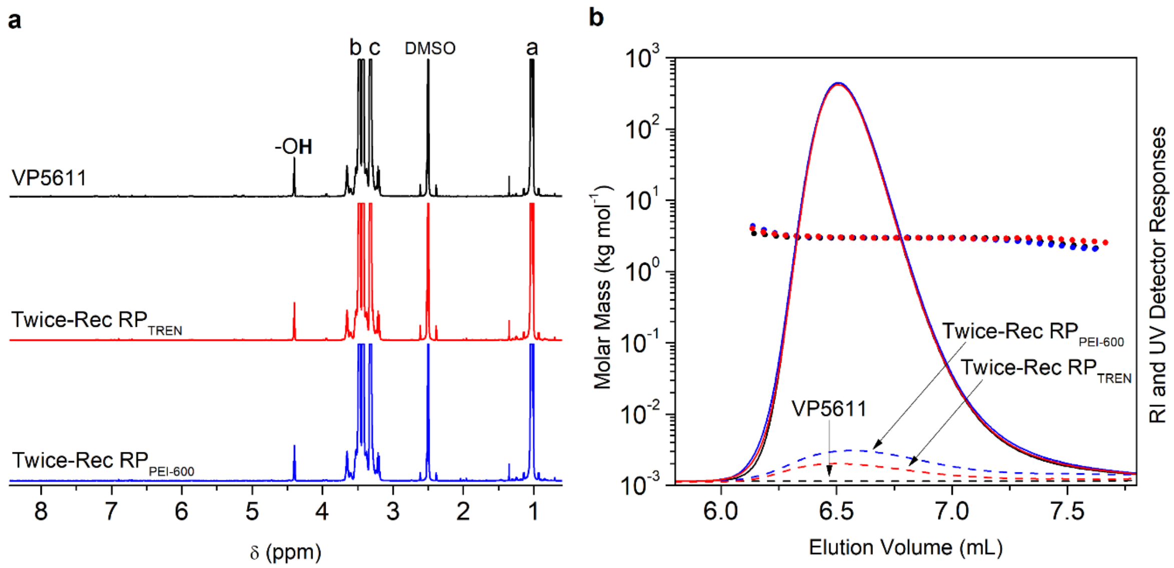

Figure S8. (a) Magnified ^{1}H NMR spectra and (b) SEC/UV-RI chromatograms of VP5611 and the corresponding twice-recycled polyols recovered from PPO-based PUF by aminolysis at 220 °C after 30 min with 13.2 wt % TREN and 13.2 wt % PEI-600. The solid and dashed curves in the SEC/UV-RI chromatograms represent the RI and UV detector responses, respectively, while the dotted lines show the molar mass as a function of elution volume.

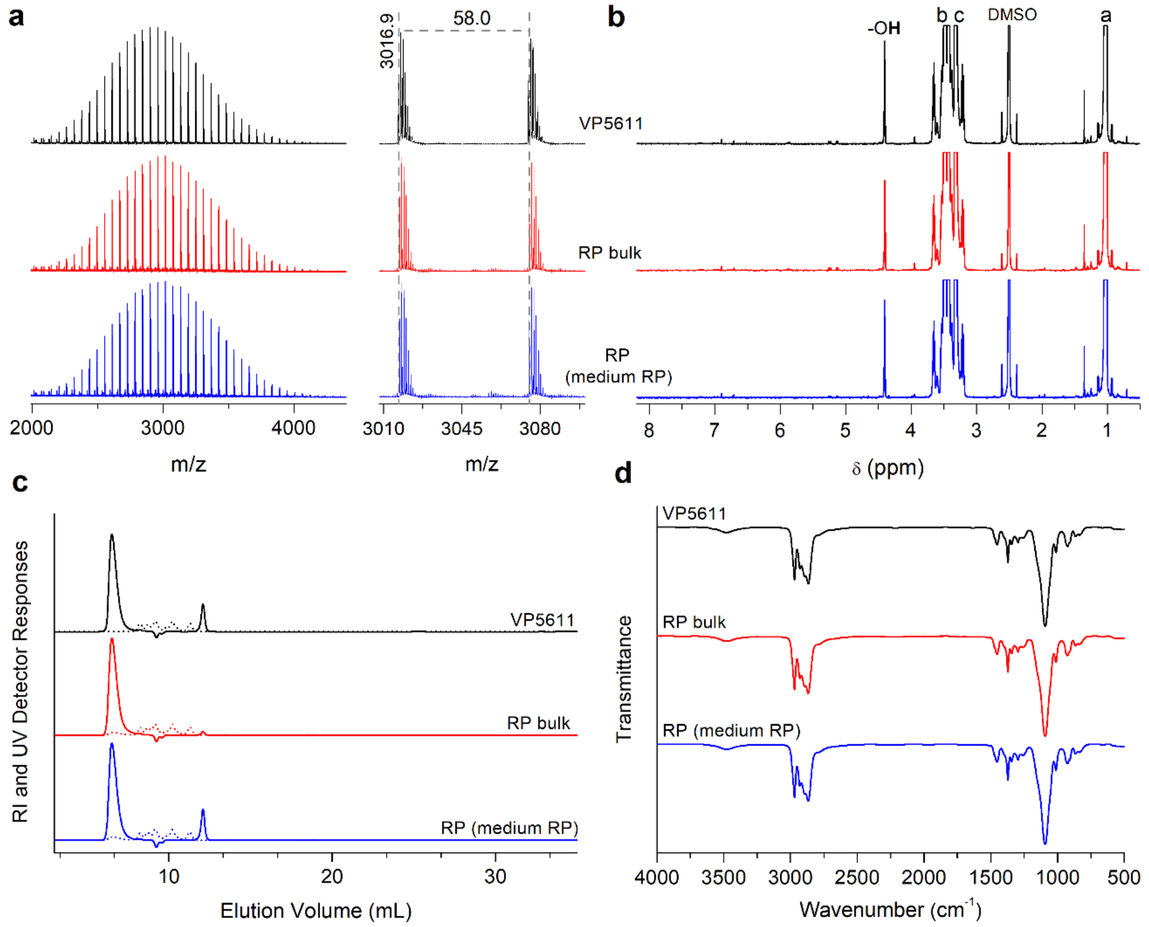

Figure S9. (a) MALDI-TOF mass spectra, (b) magnified ^{1}H NMR spectra, (c) SEC/UV-RI chromatograms and d) FTIR spectra of VP5611 and the corresponding purified RPs obtained with 13.2 wt % TREN using RP as a medium and without the use of any medium (bulk) at 220 °C after 30 min. The ^{1}H NMR spectra were normalized to the signal of methyl group of the PO repeating units. The solid and dashed curves in the SEC/UV-RI chromatograms represent the RI and UV detector responses, respectively.

Characteristics of homopolyether polyols used for the synthesis of flexible PUFs

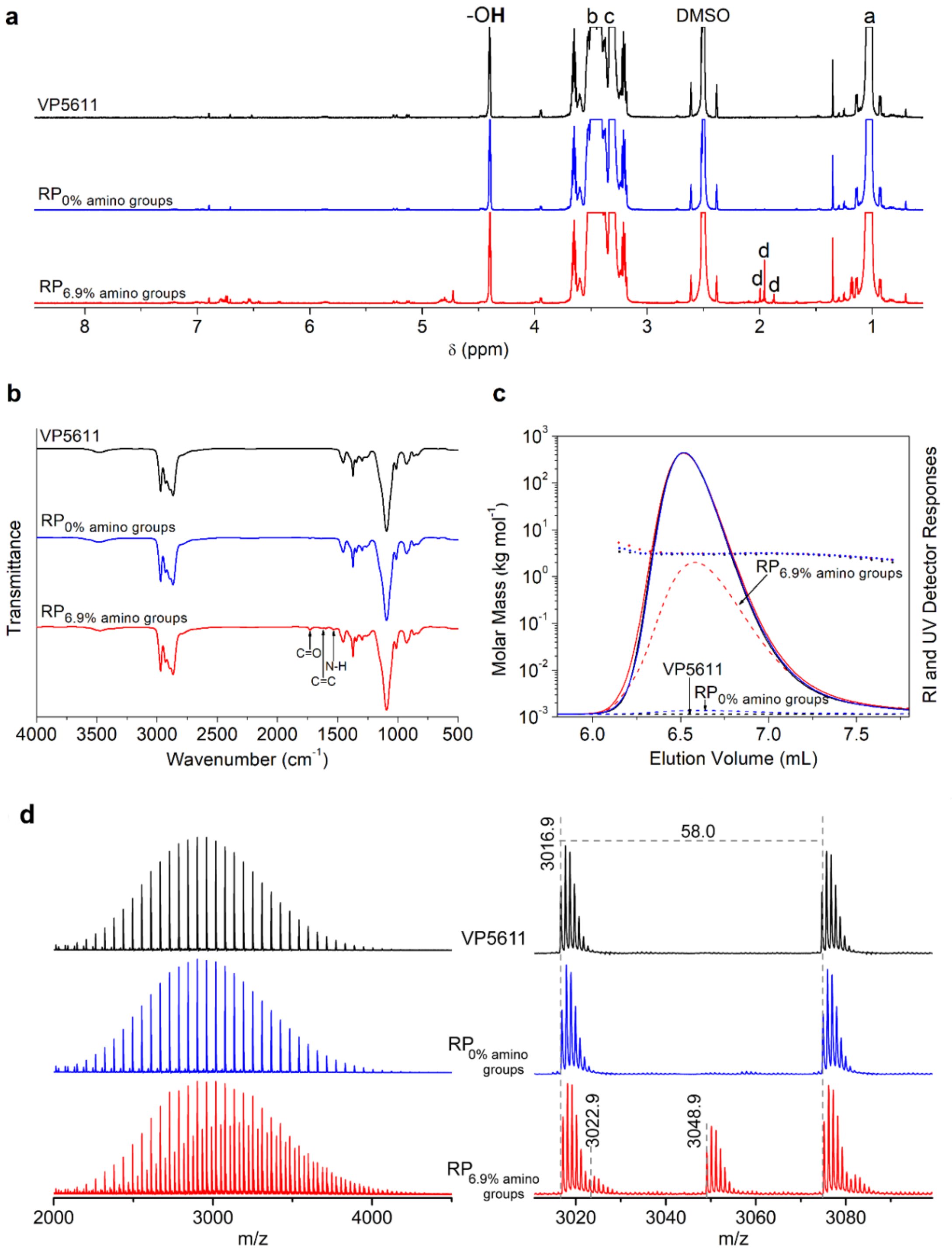

Figure S10. Magnified (a) ^{1}H NMR spectra, (b) FTIR spectra, (c) SEC/UV-RI chromatograms and (d) MALDI-TOF mass spectra of VP5611 and the corresponding RPs containing 6.9 mol % and 0.0 mol % aromatic amino end groups, recovered from PPO-based PUF by one- and two-step aminolysis with TREN, respectively. The solid and dashed curves in the SEC/UV-RI chromatograms represent the RI and UV detector responses, respectively, while the dotted lines show the molar mass as a function of elution volume.

Table S4. Properties of VP5611 and corresponding RPs recovered from PPO-based PUF by one- and two-step aminolysis with TREN.

Polyol	OH number (mg KOH g^{-1})	Acid value (mg KOH g^{-1})	Corrected OH number (mg KOH g^{-1})	Water content (%)	$-NH_2$ content[a] (mol %)	M_w [b] (kg mol^{-1})	M_w/M_n [b]
VP5611	55.97	0.05	56.02	0.029	/	3.0	1.02
$RP_{6.9\%\ amino\ groups}$	57.57	0.02	57.59	0.035	6.9	3.1	1.02
$RP_{0\%\ amino\ groups}$	53.13	0.09	53.24	0.019	0.0	3.0	1.02

[a] Determined by ^{1}H NMR spectroscopy.
[b] Determined by SEC/MALS-RI.

Flexible PUFs synthesized from homopolyether polyols

Table S5. Formulations used for PUF5611 synthesis. The amounts of the formulation components are given in “parts per hundred polyol” (pphp; w/w). The amount of PPO-based polyol used for a single PUF synthesis was 16 g.

PUF type	VP	RP	dH_2O	B11	Kosmos® 29	Silicone BF 2470	TDI 80/20	TDI index
VP5611	100	0	4.5	0.15	0.18	1.2	56.16	107
$RP_{6.9\%\ amino\ groups}$	0	100	4.5	0.15	0.18	1.2	56.16	107
$RP_{0\%\ amino\ groups}$	0	100	4.5	0.15	0.18	1.2	56.16	107

Table S6. Characteristic times during synthesis of PPO-based PUFs.

PUF type	Cream time (s)	Gel time (min)	Rise time (min)	Tack free time (h)
VP5611	20	2.38	1.50	2.25
$RP_{6.9\%\ amino\ groups}$	19	2.08	1.48	2.25
$RP_{0\%\ amino\ groups}$	22	2.48	1.56	2.15

The characteristic times were determined in accordance with ASTM D7487-18. The cream time is the time between the starting mixture and the point at which fine bubbles begin to appear; the gel time is the time at which the surface of the foam forms ‘strings’ of tacky material when touched with the spatula; the rise time is the time at which the synthesized foam stops expanding (observed visually); the tack-free time is the time at which the synthesized foam is no longer sticky when touched.

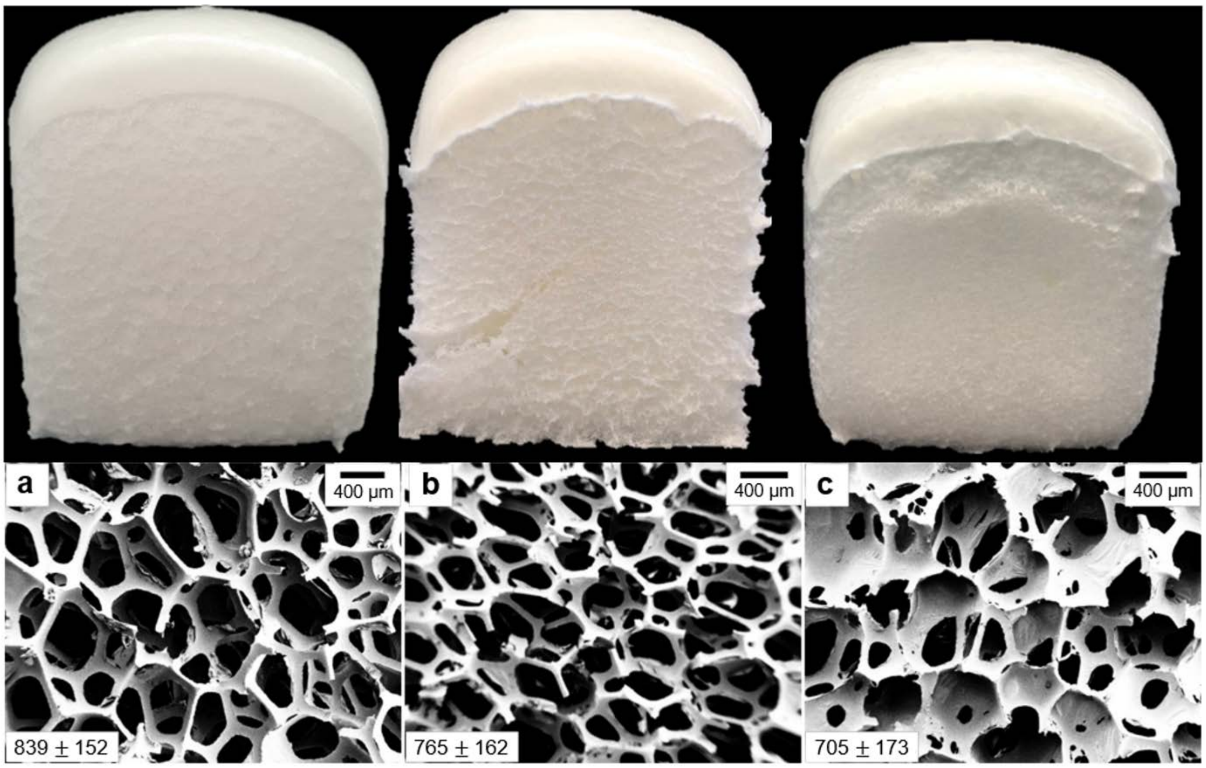

Figure S11. Photos and SEM cross-sectional images of PUFs prepared according to the formulation in Table S5 with (a) 100% VP5611, (b) 100% $RP_{0\%\ amino\ groups}$ (obtained by two-step aminolysis with TREN), (c) 100% $RP_{6.9\%\ amino\ groups}$ (obtained by one-step aminolysis with TREN). Average pore sizes in μm are given in the lower left corners of the SEM images.

Table S7. Mechanical properties of PPO-based PUFs.

**PUF Type**	**Density (kg m^{-3})**	**Compressive modulus (kPa)**	**Stress at 40% strain (kPa)**	**Compression set (%)**
VP5611	27.1 ± 1.8	16.0 ± 2.5	1.74 ± 0.22	4.0 ± 1.8
$RP_{6.9\%\ amino\ groups}$	25.4 ± 0.9	25.7 ± 5.9	2.54 ± 0.28	7.5 ± 1.9
$RP_{0\%\ amino\ groups}$	25.7 ± 1.1	14.1 ± 3.2	1.60 ± 0.18	4.6 ± 2.1

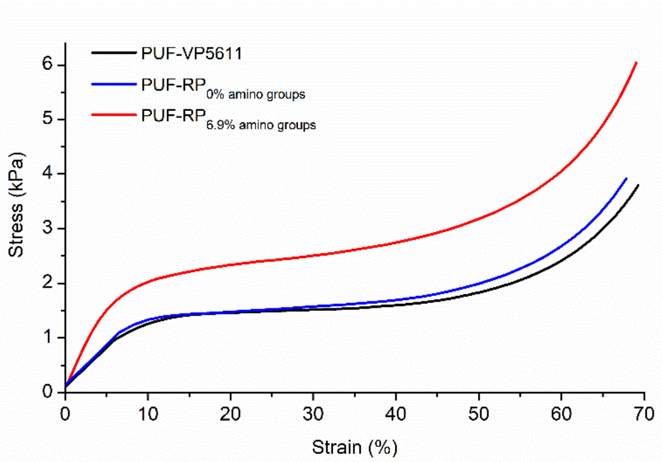

Figure S12. Compressive stress-strain curves of PPO-based PUFs prepared from 100% VP5611, 100% $RP_{0\%\ amino\ groups}$ (obtained by two-step aminolysis with TREN) and 100% $RP_{6.9\%\ amino\ groups}$ (obtained by one-step aminolysis with TREN).

Characteristics of copolyether polyols used for the synthesis of flexible PUFs

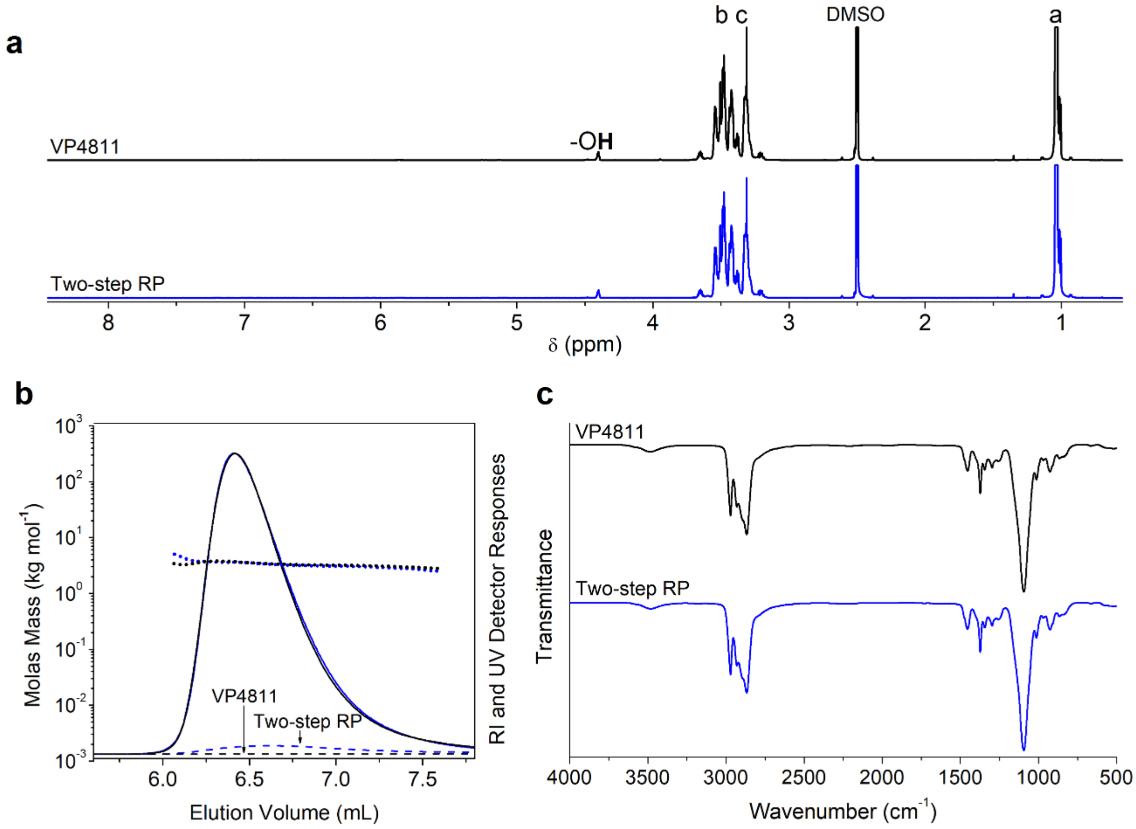

Figure S13. (a) ^{1}H NMR spectra, (b) SEC/UV-RI chromatograms and (c) FTIR spectra of fully hydroxyl-functionalized copolyether-based RP obtained by two-step aminolysis of P(PO-*co*-EO)-based PUF4811 with TREN and the corresponding virgin polyol of the same type (VP4811). The ^{1}H NMR spectra were normalized to the signal methyl group of the PO repeating units. The solid and dashed curves in the SEC/UV-RI chromatograms represent the RI and UV detector responses, respectively, while the dotted lines show the molar mass as a function of elution volume.

Table S8. Properties of VP4811 and corresponding $RP_{0\%\ amino\ groups}$ recovered from P(PO-*co*-EO)-based PUF4811 by two-step aminolysis with TREN.

Polyol	OH number (mg KOH g^{-1})	Acid value (mg KOH g^{-1})	Corrected OH number (mg KOH g^{-1})	Water content (wt %)	$-NH_2$ content[a] (mol %)	M_w[b] (kg mol^{-1})	M_w/M_n[b]
VP4811	47.68	0.05	47.73	0.018	0.0	3.5	1.02
$RP_{0\%\ amino\ groups}$	47.47	0.03	47.50	0.015	0.0	3.5	1.02

[a] Determined by ^{1}H NMR spectroscopy.
[b] Determined by SEC/MALS-RI.

Flexible PUFs synthesized from copolyether polyols

Table S9. Formulations used for PUF4811 synthesis. The amounts of the formulation components are given in "parts per hundred polyol" (pphp; w/w). The amount of polyol used for a single PUF synthesis was 100 g.

PUF type	VP	RP	dH_2O	TEGOAMIN® BDE	TEGOAMIN® 33	Kosmos® 29	Silicone L-620 LV	TDI 80/20	TDI index
1	100	0	4.0	0.10	0.115	0.23	1.25	49.42	106
2	80	20	4.0	0.10	0.115	0.23	1.25	49.38	106
3	50	50	4.0	0.10	0.115	0.23	1.25	49.33	106
4	0	100	4.0	0.10	0.115	0.23	1.25	49.23	106

Table S10. Characteristic times during synthesis of P(PO-*co*-EO)-based PUFs.

PUF number	RP content (%)	Cream time (s)	Reactivity (rise time latex/rise time reference) (Ratio < 1 more reactive; Ratio > 1 less reactive)	Rise time (s)
1	0	12	1.00	87
2	20	12	1.01	88
3	50	10	0.95	83
4	100	9	0.97	84
